# Differential Transcriptional Profiling of Damaged and Intact Adjacent Dorsal Root Ganglia Neurons in Neuropathic Pain

**DOI:** 10.1371/journal.pone.0123342

**Published:** 2015-04-16

**Authors:** A. K. Reinhold, L. Batti, D. Bilbao, A. Buness, H. L. Rittner, P. A. Heppenstall

**Affiliations:** 1 European Molecular Biology Laboratory, Monterotondo, Italy; 2 Department of Anaesthesiology, University Hospital, Würzburg, Germany; University of Texas at Dallas, UNITED STATES

## Abstract

Neuropathic pain, caused by a lesion in the somatosensory system, is a severely impairing mostly chronic disease. While its underlying molecular mechanisms are not thoroughly understood, neuroimmune interactions as well as changes in the pain pathway such as sensitization of nociceptors have been implicated. It has been shown that not only are different cell types involved in generation and maintenance of neuropathic pain, like neurons, immune and glial cells, but, also, intact adjacent neurons are relevant to the process. Here, we describe an experimental approach to discriminate damaged from intact adjacent neurons in the same dorsal root ganglion (DRG) using differential fluorescent neuronal labelling and fluorescence-activated cell sorting (FACS). Two fluorescent tracers, Fluoroemerald (FE) and 1-dioctadecyl-3,3,3,3-tetramethylindocarbocyanine perchlorate (DiI), were used, whose properties allow us to distinguish between damaged and intact neurons. Subsequent sorting permitted transcriptional analysis of both groups. Results and qPCR validation show a strong regulation in damaged neurons versus contralateral controls as well as a moderate regulation in adjacent neurons. Data for damaged neurons reveal an mRNA expression pattern consistent with established upregulated genes like galanin, which supports our approach. Moreover, novel genes were found strongly regulated such as corticotropin-releasing hormone (CRH), providing novel targets for further research. Differential fluorescent neuronal labelling and sorting allows for a clear distinction between primarily damaged neuropathic neurons and “bystanders,” thereby facilitating a more detailed understanding of their respective roles in neuropathic processes in the DRG.

## Introduction

Neuropathic pain is defined as pain arising from a lesion within the somatosensory system. This includes nerve injury, central neuropathic pain as well as peripheral polyneuropathies. Treatment of neuropathic pain remains a challenge for clinicians. Currently, anticonvulsants and antidepressants are commonly used but a significant number of patients cannot achieve sufficient pain relief [1]. To close this therapeutic gap and identify new pharmaceutical targets, a better understanding of underlying processes is necessary. Yet, cellular and molecular mechanisms of neuropathic pain are complex and may vary considerably. Changes in gene expression include neuropeptides (galanin, neuropeptide y), ion channels (voltage-gated channels, purinergic channels), and genes involved in apoptosis and stress response, such as Atf3 after axotomy of the sciatic nerve [2,3]. Moreover, they vary among tissues: In addition to the nervous system, also the immune system is critical to the pathophysiology of neuropathic pain. A pathological neuro-immune communication has also been associated with painful neuropathy [4,5]. This complexity is well reflected by the tissue heterogeneity in DRG: Studies suggest that only 15% of all DRG cells are neurons [6]. The largest numbers of other cells include glia, i.e. Schwann and satellite cells. As the proportions of neurons versus glia fluctuate across DRGs, cell-type-specific expression changes may vary considerably and, moreover, be masked by high background signal. While whole-DRG approaches are established in the study of peripheral neuropathies [7,8], these do not distinct between different cell populations and their contribution. Yet, it is precisely this heterogeneity of cells in the DRG that might cause limitations in the investigation of transcriptional regulation after injury [9].

On a neuronal level, gene regulation not only occurs in primarily damaged neurons but also in adjacent intact neurons: For example, intact nociceptors become sensitized to adrenergic agents as well as to tumor necrosis factor-α (TNF-α) [10,11]. Moreover, an overexpression of transient potential receptor V1 (TRPV1) and voltage-gated sodium channels has been observed in spared dorsal root ganglia (DRG) neurons after ligation [12]. However, most research on “uninjured afferent” neurons [13] originate from comparisons between injured and not-injured DRG (e.g. L4 after spinal nerve ligation (SNL) of L5, or spared nerve branches after partial ligation, see [14]) rather than neighboring neurons of the same DRG.

Fluorescent neuronal labelling has been established to identify neuronal subsets. However, these studies did not distinct between injured and uninjured neurons. Double-labelling *in vivo* for injured versus spared neurons (e.g. fluororuby and fluorogold, [10]) has so far mainly been used for immunohistochemistry. By combining differential fluorescent neuronal labelling with fluorescence-activated cell sorting (FACS), we now developed an approach that not only allows the study of neuron-specific expression but also enabled us to compare gene expression in damaged and adjacent intact DRG neurons after chronic constriction injury (CCI). Fluoroemerald (FE) is a fluorescein-labelled 10,000 Da dextran [15]. As its high molecular weight impedes the permeation of intact neuronal membranes, it can be taken up only by neurons with an impaired membrane barrier function and is therefore suitable for the labelling of damaged neurons [16]. In contrast, 1-dioctadecyl-3,3,3,3-tetramethylindocarbocyanine perchlorate (DiI), an amphiphilic carbocyanine with two long hydrocarbon side chains, is quickly taken up by neurons and embedded in the lipid bilayer of the cell membrane where it passively diffuses along the axon [17]. DiI is therefore a good marker for neurons [18,19]. Thus, through FACS, a selection and separation of damaged (FE+) and intact (FE-) neurons is viable. The distance between the application site and the soma (DRG) prevents accidental selection of non-neuronal tissue. By combining differential fluorescent neuronal labelling with FACS, we developed an approach that not only allowed us to obtain neuron-specific expression patterns but to also compare gene expression in damaged and adjacent intact DRG neurons in mice with chronic constriction neuropathy.

## Materials and Methods

### Animals and CCI

Female C57/BL6 mice of 6–8 weeks of age were used (Charles River Laboratories, Wilmington, MA, USA). Mice were housed in sawdust cages (4–5 mice per cage, with water and food provided ad libitum). Mice were anesthetized with an intraperitoneal injection of 2.5% tribromoethanol (Sigma Aldrich, St Louis, MO, USA) or isoflurane 1.5 Vol% (Baxter, Deerfield, IL, USA). The sciatic nerve was located and exposed after skin incision. Three friction-knotted loose ligations were tied around the sciatic nerve using 7–0 silk threads and the wound was closed with a metal clip [20]. Sham surgery consisted of anaesthesia and exposure of the nerve but without nerve ligation. For euthanasia, cervical dislocation was used. All efforts were made to minimize suffering. Animal experiments were approved by EMBL Monterotondo Animal Committee and comply with Italian legislation (Art. 9, 27. Jan 1992, no 116, under licence from the Italian Ministry of Health).

### Neuronal labelling

**Fig 1:**
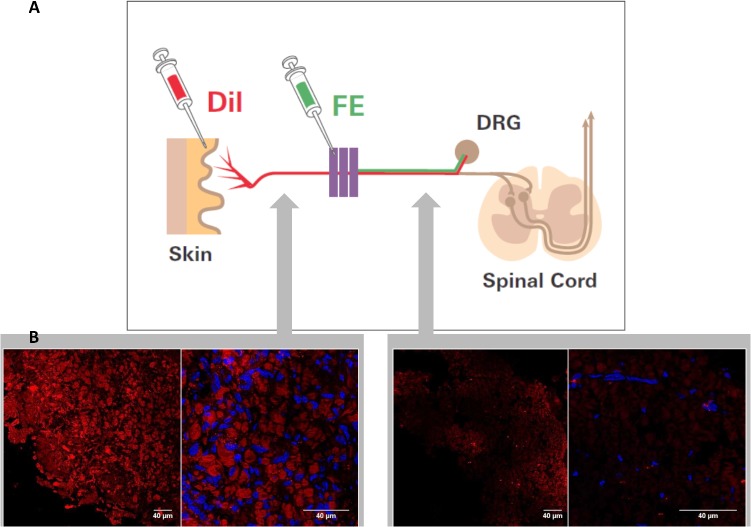
Principle of fluorescent tracer injection and DiI signal after CCI. (**A**) Fluoroemerald (FE, green) is applied proximal to the site of injury; it is taken up by damaged neurons and transported to the DRG. DiI (red) is injected into the hindpaw immediately after the surgical procedure. It permeates the axonal membrane and diffuses along the axon. Membrane disruption, however, impedes further diffusion towards the DRG. (**B**). One week after CCI, the sciatic nerve was excised and cryosected to examine native DiI intensity. Compared to the distal sciatic nerve (left panel), transections proximal to the site of injury (right panel) exhibit a clearly decreased DiI intensity. Nuclei were visualized with blue DAPI (n = 4, representative sample, scale bar = 40 μm).

Immediately following CCI ligation, 2 μl of FE (5% in 0.9% saline, Invitrogen, Carlsbad, CA, USA) were injected epineurally into the exposed nerve proximal to the ligation site using a Hamilton syringe with a 32 G needle or micropipette. Great care was taken not to penetrate deeper layers of the nerve. After closure of the wound, 4 μl DiI (10 mg/ml in dimethyl sulfoxide (DMSO), Carlsbad, CA, USA) were injected subcutaneously into the plantar surface of both hindpaws using a 28 G needle (**Fig 1A**). The site of injection was manually pressed for one minute to facilitate puncture closure and avoid dye leakage.

### Tissue harvest

After 7 d, mice were sacrificed by cervical dislocation. The proximal parts of the sciatic nerve were exposed and traced back to the respective spinal nerves. The corresponding DRG (L3-5) were excised, detached from axons and surrounding tissue. DRG cells were isolated after treatment with 1 mg/ml collagenase IV in Dulbecco’s modified eagle medium (DMEM) and 0.05% trypsin in ethylenediaminetetraacidic acid (EDTA) for 25 and 22 min, respectively, at 37°C. Resuspended in DRG medium (10% horse serum heat-inactivated, 100 μg penicillin, 100 μg/ml streptomycin, 0.8% glucose in DMEM), cells were triturated and filtered. Immediately before cell sorting, SytoxBlue (Invitrogen, Carlsbad, CA, USA), was added to control for viability. For qPCR analysis, a mouse monoclonal anti-CD45 Ab (PECy7, 1:200) was added for 10 min to label and later exclude haematopoietic cells. With two neuronal tracers applied ipsilaterally, four tracer combinations were possible for cell staining (**Table 1**). From ipsilateral neurons, two populations were sorted: FE^+^ (damaged by CCI), and FE^-^/DiI^+^ cells (neurons projecting from the hind paw but not damaged by CCI). The four-colour flow cytometric analysis was carried out with a five-laser FACS Aria SORP (BD, Heidelberg, Germany); bandpass filters for detection of the different dyes were 530/30 for FE, 582/15 for DiI, 780/60 for PE-Cy7, and 450/50 for Sytox Blue. For microarray analysis, each run consisted of DRGs pooled from 12 mice; for qPCR, DRGs were pooled from 4 mice per run.

**Table 1:** Tracer combinations and their interpretation.

FE staining	DiI staining	Interpretation
**+**	**+**	Sensory neuron (hind paw afferent), partially damaged
**+**	**-**	Sensory neuron, damaged
**-**	**+**	Sensory neuron (hind paw afferent), not damaged
**-**	**-**	Any but the above

DiI: 1-dioctadecyl-3,3,3,3-tetramethylindocarbocyanine perchlorate; FE: Fluoroemerald

### RNA extraction and assay

Tissue homogenization and RNA extraction followed the standard Trizol protocol (Invitrogen, Carlsbad, CA, USA). RNA quantity and quality were assessed by Nanodrop 8000 (Nanodrop Technologies, Wilmington, DE, USA) and Agilent 2100 Bioanalyzer (Agilent Technologies, Santa Clara, CA, USA) respectively. Isolated RNA was stored at -80°C and shipped to EMBL Heidelberg for Affymetrix Gene Expression analysis (assay: *Affymetrix Gene Expression Mouse 430_2;* conducted by Sabine Schmidt, EMBL Heidelberg). Three runs were performed, each with sorted cells from 12 mice.

### qPCR

For 40 selected genes, qPCR was conducted. Primers were designed using PrimerBLAST engine (http://www.ncbi.nlm.nih.gov/tools/primer-blast/index.cgi, manufactured by Eurofins, http://www.eurofinsgenomics.eu/. For p0072imer sequences, see **S1 Table**). As reference genes served Advillin, GAPDH, and Ubiquitin C. Reverse Transcription and pre-amplification were carried out immediately after flow cytometry using CellsDirect OneStep qRT PCR Kit (Invitrogen, Carlsbad, CA, USA) and cDNA then shipped to EMBL Gene Core facility for qPCR using Fluidigm technology (conducted by Paul Collier, EMBL Heidelberg). Two runs were performed, each with sorted cells from 4 mice.

cDNA was analyzed using the ΔΔCt normalization method. Normalization was based on the mean Ct value of the three reference genes (ΔCt), followed by normalization based on the contralateral ΔCt value (ΔΔCt).

### Nerve preparation

After sacrification, the sciatic nerve (trifurcation to spinal nerve branching) was excised, briefly washed in phosphate-buffered saline (PBS) and fixed in 4% paraformaldehyde (PFA) for 2 h at 4°C. After embedding in TissueTek OCT compound (Sakura, Torrance, CA, USA), transverse sections of the nerve (10 μm), proximal and distal to the site of injury, were cut. Nuclei were stained with 4′,6-Diamidin-2-phenylindol (DAPI, 1:1000) for 5 min and sections embedded in ProLong Gold mounting medium (Life Technologies, Darmstadt, Germany). Images were acquired using a Leica DMR microscope (Leica, Wetzlar, Germany).

### Immunohistochemistry

Anaesthetized animals were perfused with 4% paraformaldehyde in phosphate buffer (PB, 0.1 M, pH 7.4) seven days after CCI or sham surgery. The L5 DRGs were fixed for 4 h in the same solution and cryoprotected overnight in 30% sucrose in PB. Transverse sections of DRG (20 μm) were cut, blocked with 10% normal donkey serum (Sigma Aldrich, St Louis, MO, USA) in PBS for 40 min, and incubated overnight with primary antibody rabbit polyclonal anti-CRH Ab (1:1000, Phoenix, Burlingame, CA, USA). Anti-rabbit IgG Alexa 488 (Invitrogen, Carlsbad, CA, USA) served as the secondary antibody. Images were acquired with a Leica DMR microscope (Leica, Wetzlar, Germany).

### Statistical analysis

Microarray data were normalized using the Robust Multi-array Average (RMA) algorithm with background correction and quantile normalization (www.bioconductor.org). A two-way ANOVA model without interaction was used to model conditions and runs. For each probe set, moderated t-statistics were calculated for all pair-wise contrasts between conditions. Multiple testing correction was done using the Benjamini-Hochberg method. Statistical significance was determined based on the false discovery rate with p < 0.05. In addition to significant genes, genes with p > 0.05, < 0.1 plus a high fold-change (> 2-fold up or down) were included into further analysis and validation steps to account for biological significance. For cluster analysis, hierarchical clustering with complete linkage was performed for 200 probe sets with highest median absolute deviation (MAD). Expression values were standardized by applying a z-score transformation per gene. Raw data were uploaded on ArrayExpress (Accession ID E-MTAB-3326)

For all expressed genes from the qPCR analysis, pairwise t-statistics between conditions were performed with statistical significance determined as p < 0.05 (SigmaPlot, Systat Inc, San Jose, CA, USA).

## Results

### Reduction of DiI signaling proximal of lesion

The tracer DiI is known to be taken up by neurons, to be integrated into membranes of intact nerve fibres and to travel retrogradely to the DRG in nociceptors. To first verify the hypothesis that DiI transport in damaged neurons is reduced after CCI and that CCI-induced damage affects considerable portions of the nerve, transections of the sciatic nerve distal and proximal to the lesion were compared for DiI signalling. 7 days after CCI, DiI intensity in the sciatic nerve proximal to the lesion is decreased greatly compared to distal sites. Moreover, morphology of stained structures support axonal staining rather than nucleated, e.g. glial (Schwann) cells **(Fig 1B)**.

### Similar numbers of damaged and adjacent intact neurons in DRGs

**Fig 2:**
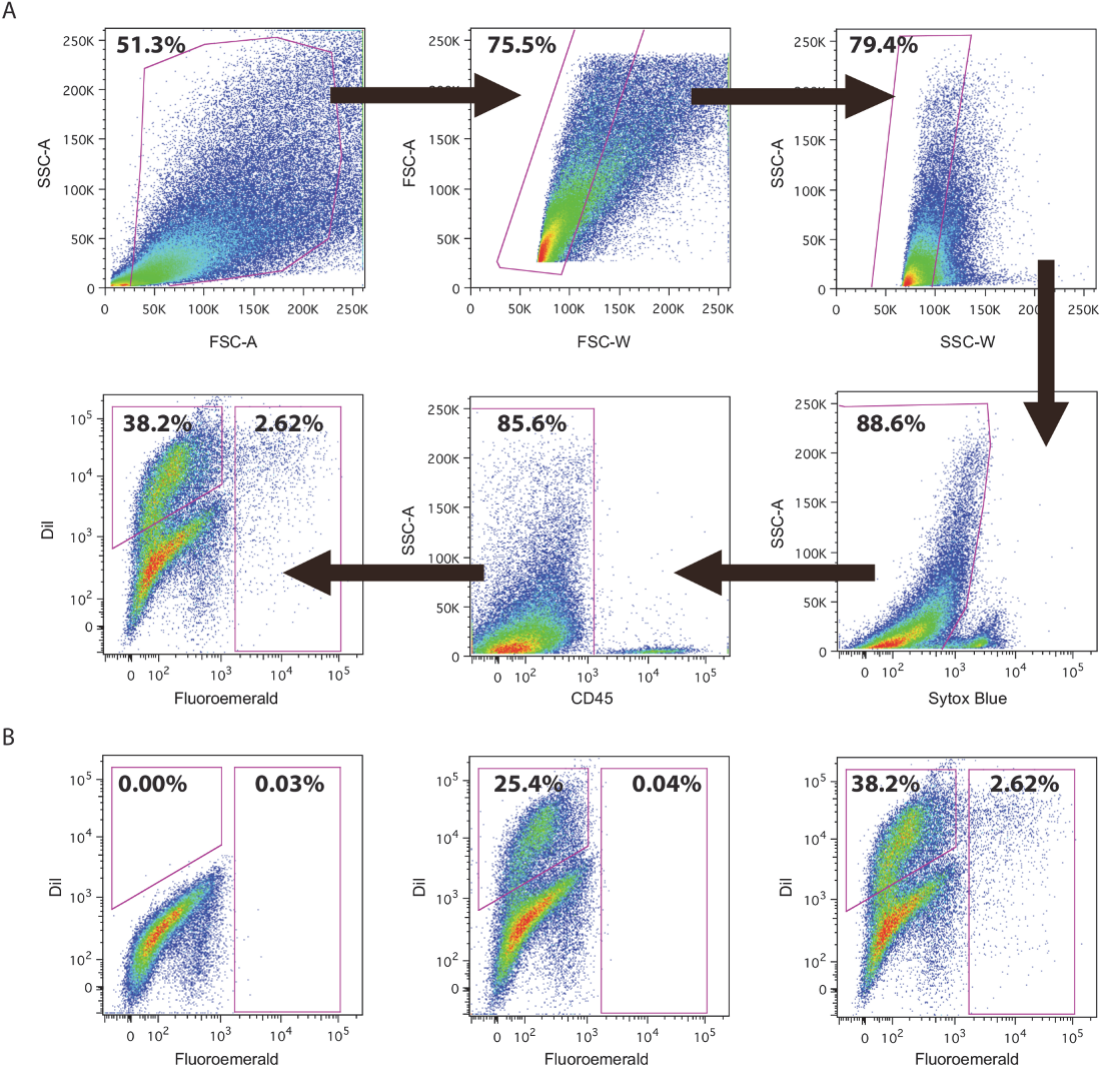
Flow cytometric detection of damaged and intact neurons for qPCR. DRGs L3-5 were harvested and cells isolated 7 days after CCI. The sorting strategy to identify neurons positive for Fluoroemerald (FE) and DiI is shown in (**A**). Initially, cells were gated for size and granularity, before excluding dead cells using Sytox Blue and haematopoetic cells using CD45-Ab Cy7. The remaining cells were sorted for DiI and FE. FACS plots of negative control (**B left**), contralateral (**B middle**) and ipsilateral (**B right**) DRG cells are displayed in the lower panel. DiI^+^/FE^-^ cells are considered to be spared neurons, FE^+^ cells are damaged neurons. Both populations were obtained for further analysis (n = 3, representative example).

In the next step we analyzed cells in the DRG regarding their uptake of the two markers DiI for intact neurons and FE for damage neurons by flow cytometry before sorting. In the initial flow cytometric analysis of single cell suspensions from mice DRG 7 d after CCI, 2.01% of viable cells included were Dil^+^/FE^-^ (adjacent intact neurons) and 1.01% of all cells were FE^+^ (consistent with damaged neurons) (**S1 Fig**). No FE^+^ cells were found contralaterally. These sorted cells were further used for the gene expression profile Percentage of DiI^+^ undamaged neuron detected varied in runs for qPCR. For samples preparation for qPCR, CD45^+^ hematopoietic cells were also excluded **(Fig 2)**.

### Consistent expression profiles for damaged and contralateral neurons

**Fig 3:**
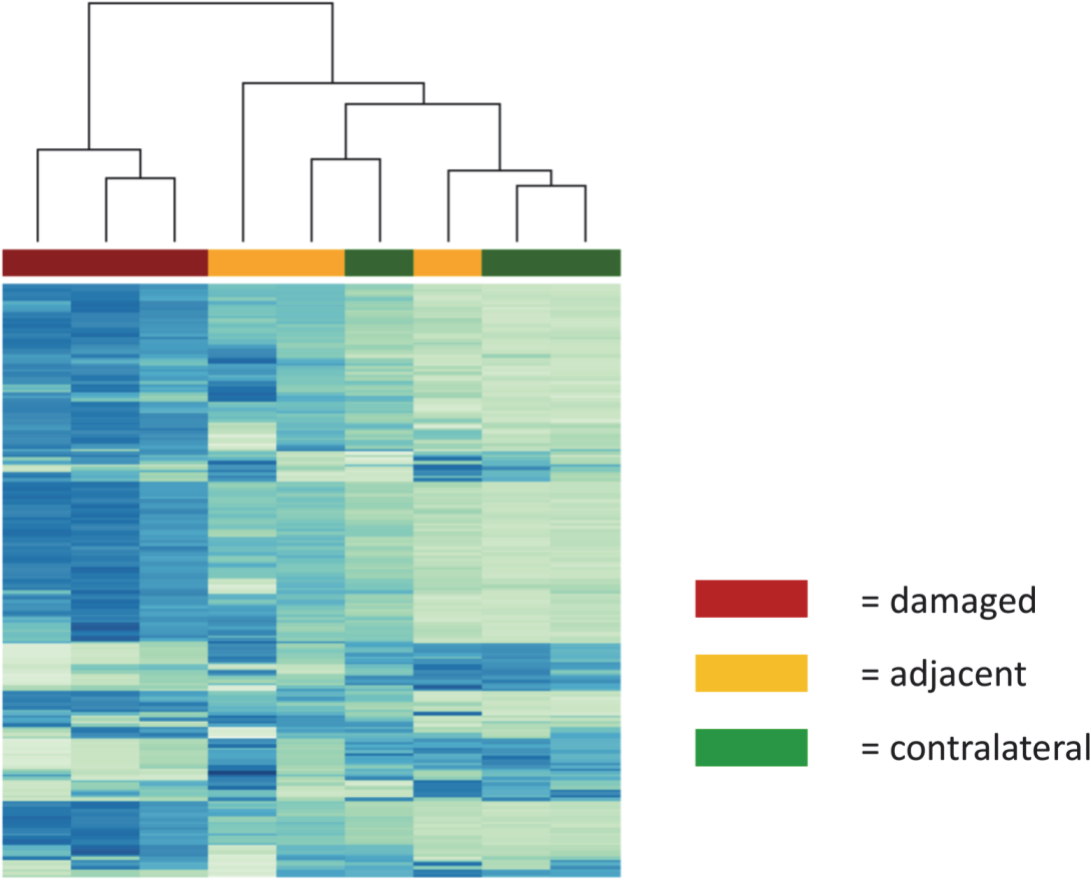
Microarray cluster analysis. Samples of damaged neurons (red) exhibit within-group similarity and considerable group difference to both adjacent (yellow) and contralateral (green) neurons. In contrast, these two groups cannot be discriminated from each other (hierarchical clustering with complete linkage for 200 probe sets with highest MAD. (Color key: row z-score -2 (dark blue) to +2 (white)).

From sorted cell populations (ipsilateral DiI^+^/FE^+^, ipsilateral DiI^+^/FE^-^ and contralateral DiI^+^ cells), RNA was purified and further analysed using the *Affymetrix* Gene Expression array. Cluster analysis of all three runs revealed a homogenous expression profile in damaged neurons Samples of adjacent, non-damaged neurons, in contrast, exhibit a broader within-group variety of gene expression (**Fig 3**).

**Fig 4:**
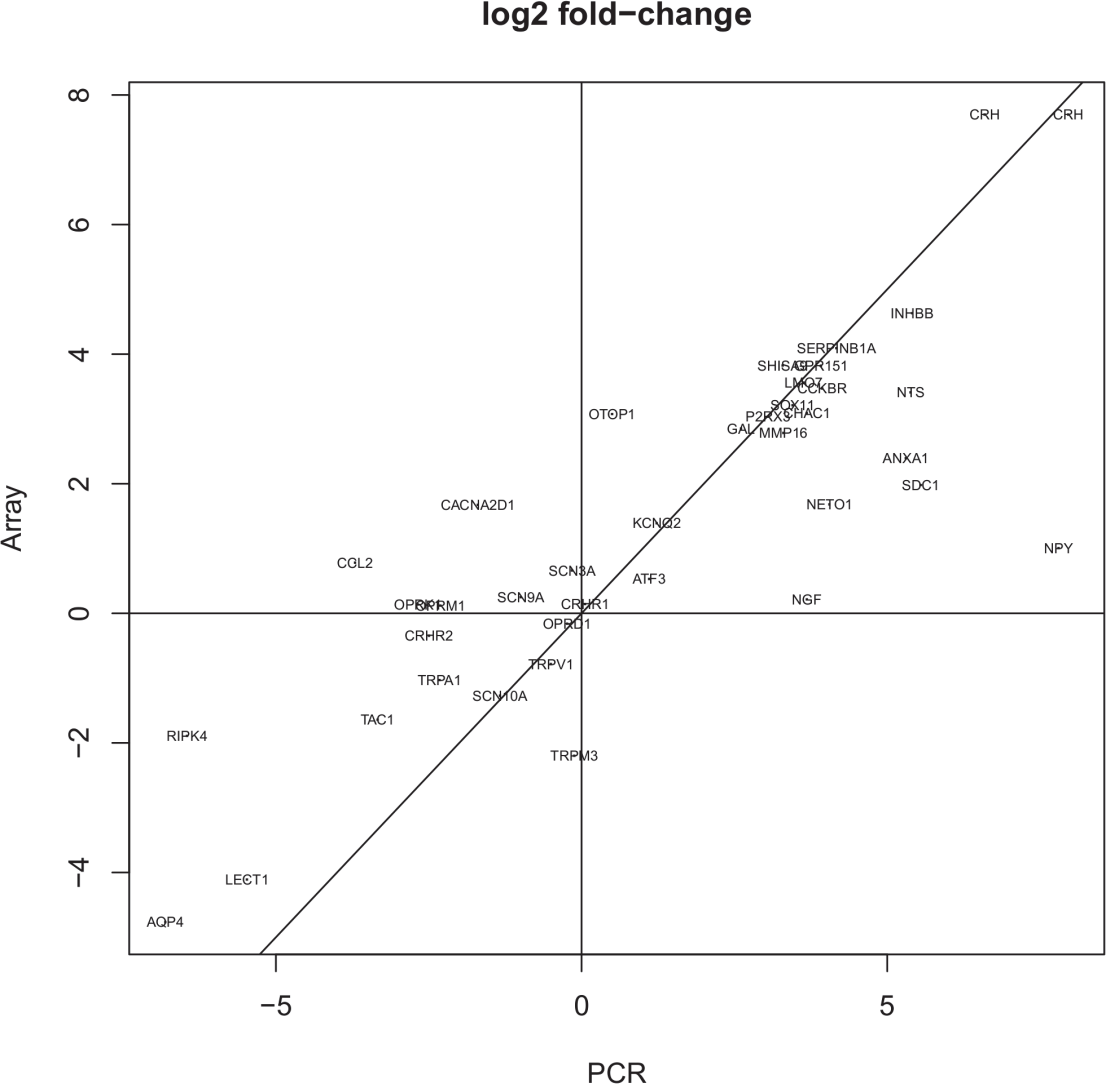
Correlation analysis between microarray and qPCR. For genes tested by qPCR, differential expression in damaged vs. contralateral neurons was compared to microarray results. The scatter plot shows a fair correlation.

Microarray analysis screened for 45,101 gene identifiers. Of these, 1172 (2.6%) were regulated in damaged compared to contralateral neurons, and 567 genes (1.3%) between damaged and adjacent neurons. Comparison between contralateral and adjacent neurons did not uncover any significant differences in the expression of selected genes. Validation for 40 genes through qPCR correlated with microarray results (**Fig 4**).

### Genes regulated in damaged versus contralateral sensory neurons

**Fig 5:**
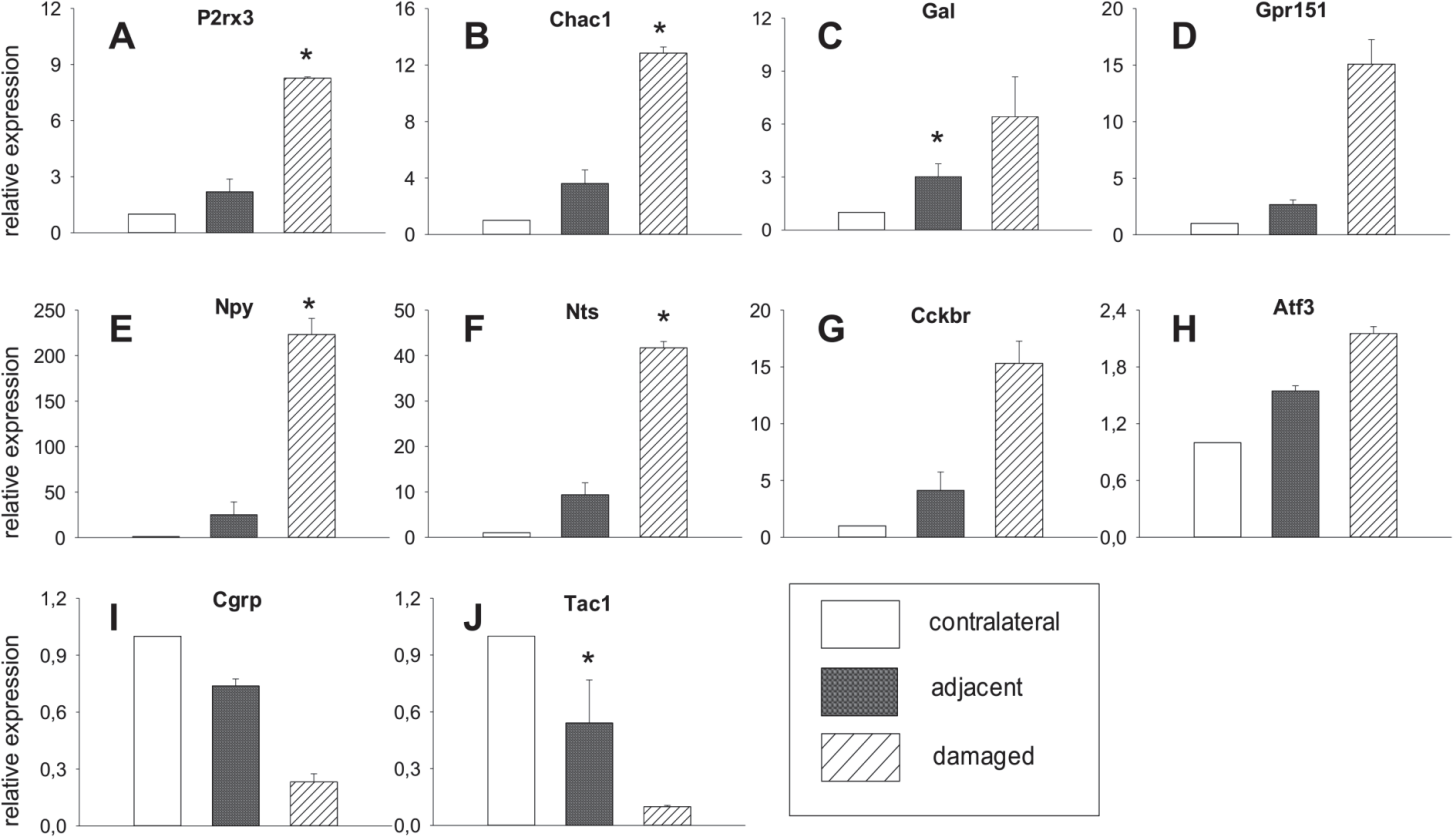
qPCR results for known genes. (**A-J**) Expression levels in damaged and adjacent neurons relative to contralateral neurons are shown for ion channels and neuropeptides previously described in neuropathic pain (mean ± SEM, n = 2, paired t-test, *p < 0.05 compared to contralateral).

**Fig 6:**
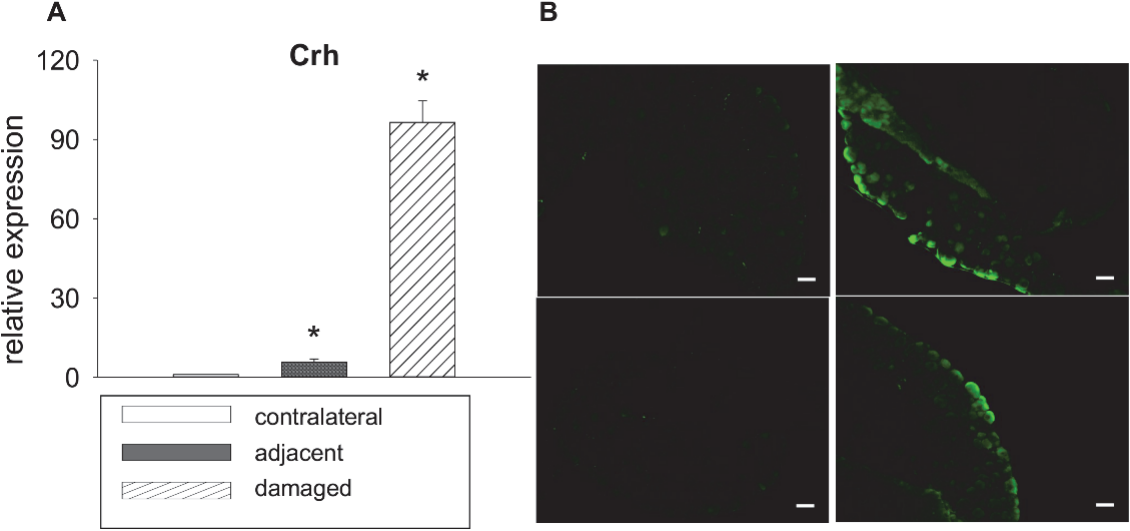
CRH expression in DRG neurons after CCI. (**A**) mRNA from DRG neurons 7 d after CCI was analyzed by qPCR, It shows a strong, significant upregulation in damaged neurons and, to a smaller degree, in adjacent neurons compared to contralateral neurons (mean ± SEM, n = 2, paired t-statistics *p < 0.05 compared to contralateral). (**B**) Immunohistochemistry for CRH in DRG neurons after CCI. Ipsilateral DRG were obtained 7 d after CCI or sham surgery and immunostained for CRH. Immunoreactivity for CRH was very low and nearly undetectable in sham controls (right panel). DRGs from neuropathic animals (left panel) show a robust immunoreactivity of CRH, detected in the cytoplasm of small, medium and large size cell bodies (scale bar: 60 αm, two representative samples, n = 3).

**Fig 7:**
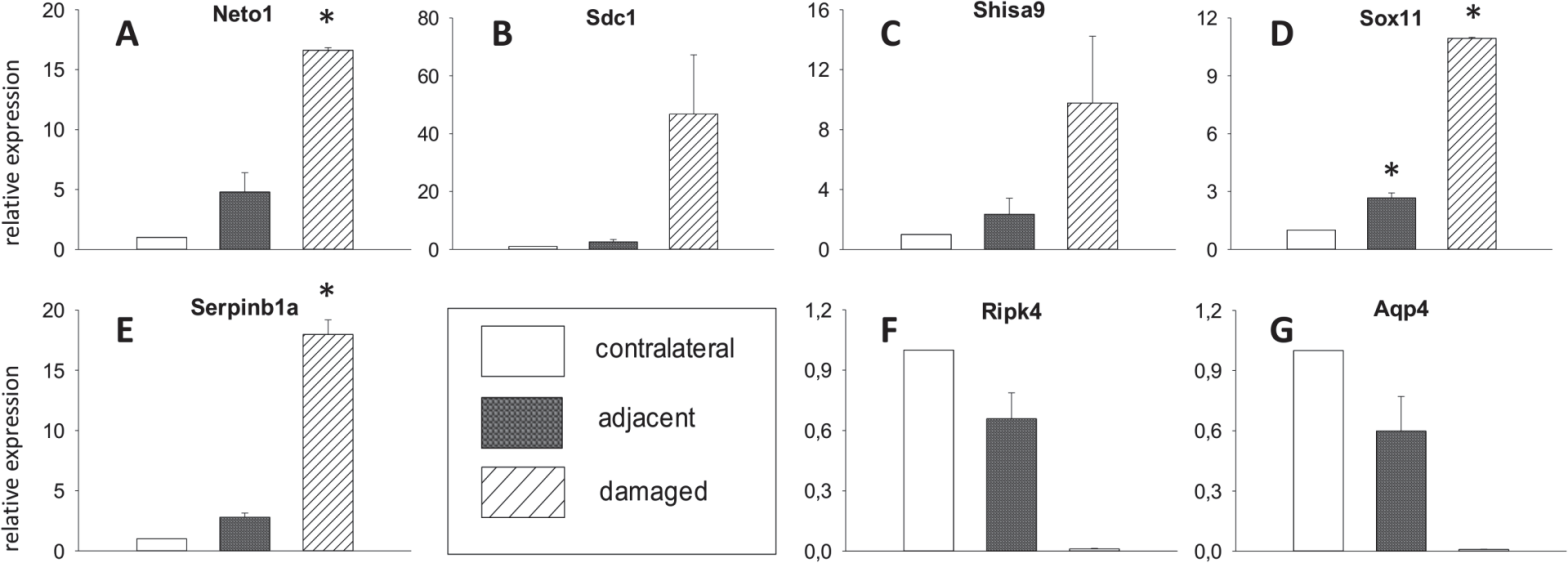
qPCR results for novel genes. (**A-G**) Expression levels in damaged and adjacent neurons relative to contralateral neurons are shown for genes not yet known in neuropathic pain (mean ± SEM, n = 2, paired t-test, *p < 0.05 compared to contralateral).

A vast number of expressed genes were significantly regulated in damaged primary sensory neurons compared to contralateral neurons (**Tables 2 and 3 and S2 Table**). Microarray showed considerable regulation for several ion channels in damaged neurons: Purinergic receptor *P2rx3*, an adenosine triphosphate(ATP)-gated ion channel was highly upregulated in damaged neurons: 8.2-fold compared to contralateral neurons (p<0.05) and 4.3-fold compared to adjacent neurons (p = 0.09). Other channels with a higher expression compared to contralateral neurons include voltage-gated calcium channel alpha 2 delta subunit 1 (*Cacnα2δ1*, 3.8-fold, p<0.05), and anoctamin 4, a calcium-activated chloride channel of the TMEM16 family (4-fold, p<0.05). For *P2rx3* as well as for cation transport regulator-like 1 (*Chac1* **, Fig 5A and 5B**), this observation was validated by qPCR. Downregulated channels include potassium channels *Kcnk2* (4-fold), *Kcnj10* (5.3-fold), *Kcnh8* (5.7-fold), and *Kcnn4* (6.6-fold, all p<0.05). Of the classical signalling neuropeptides known to be involved in neuropathic pain, galanin expression was considerably augmented in damaged neurons (7.2-fold vs. contralateral neurons, 3.7-fold vs. adjacent neurons, p<0.05), as well as its receptor *Gpr151* (14-fold vs. contralateral, p<0.05, 7.8-fold vs. adjacent, p = 0.07, **Fig 5C and 5D**). Both results were validated through PCR which also confirmed upregulation of neurotensin, neuropeptide Y, cholecystokinin receptor B and transcription factor *Atf3* (**Fig 5E–5H**). Neuropeptides with a decreased expression in qPCR (though not significant in the microarray analysis) included calcitonin gene-related peptide (*Cgrp*) and substance P/tachykinin (*Tac1*, **Fig 5I and 5J**). The most-upregulated gene was corticotropin releasing hormone *(Crh*). Its striking overexpression in damaged neurons (<200-fold vs. contralateral, p = 0.06) was also validated by qPCR **(Fig 6A)**. Similarly, expression levels were elevated for kainate receptor-modulator neuropilin and tolloid-like 1 (*Neto1*, 19-fold vs. contralateral, p = 0.08), syndecan 1 (10-fold vs. contralateral, p = 0.1), AMPAR-associated *Shisa9* (14.2-fold vs. contralateral, p<0.05, 11.7-fold vs. adjacent, p = 0.05), SRY box-containing gene 11 (*Sox11)* (10.7-fold vs. contralateral, p<0.05, 10.5-fold vs. adjacent, p = 0.05), and peptidase inhibitor *Serpinb1a* (17-fold vs. contralateral, p<0.05, 11.7-fold vs. adjacent, p = 0.08), all of which were confirmed by qPCR **(Fig 7A and 7E)**. Prominently downregulated were genes encoding for water channel aquaporin 4 (-27.1-fold vs. contralateral, p<0.05, -18.3-fold vs. adjacent, p = 0.06) and *Ripk4* (-27,3-fold vs. contralateral, p<0.05, **Fig 7F and 7G)**.

**Table 2:** Genes upregulated in damaged DRG neurons compared to contralateral control.

*Symbol*	*Gene name*	*adjusted p*	*upregulation*
*Crh*	corticotropin releasing hormone	0.064	207.7
*Sprr1a*	small proline-rich protein 1A	0.091	34.5
*Inhbb*	inhibin beta-B	0.024	24.8
*Neto1*	neuropilin (NRP) and tolloid (TLL)-like 1	0.081	19.9
*Serpinb1a*	serine peptidase inhibitor, clade B, member 1a	0.033	17.1
*Shisa9*	shisa homolog 9 (Xenopus laevis)	0.020	14.2
*Gpr151*	G protein-coupled receptor 151	0.023	14.1
*Lmo7*	LIM domain only 7	0.023	11.8
*Cckbr*	cholecystokinin B receptor	0.039	11.1
*Sdc1*	syndecan 1	0.099	10.7
*Sox11*	SRY (sex determining region Y)-box 11	0.022	10.7
*Nts*	neurotensin	0.070	10.6
*Mmp16*	matrix metallopeptidase 16	0.050	10.6
*Chac1*	ChaC, cation transport regulator 1	0.062	8.5
*Otop1*	otopetrin 1	0.063	8.4
*P2rx3*	purinergic receptor P2X, ligand-gated ion channel, 3	0.025	8.2
*Sez6l*	seizure related 6 homolog like	0.024	7.9
*Bcat1*	branched chain aminotransferase 1, cytosolic	0.093	7.4
*Gal*	galanin	0.010	7.2
*Vgf*	VGF nerve growth factor inducible	0.055	4.0
*Cd109*	CD109 antigen	0.024	4.0
*Ano4*	anoctamin 4	0.024	4.0

(n = 3, two-way ANOVA with Bejamini-Hochberg correction. Selected genes, all p < 0.1 plus fold change >2)

**Table 3:** Genes downregulated in damaged DRG neurons compared to contralateral control.

*Symbol*	*Gene name*	*adjusted p*	*downregulation*
*Ripk4*	receptor-interacting serine-threonine kinase 4	0.027	27.29
*Aqp4*	aquaporin 4	0.022	27.20
*Bcan*	Brevican	0.010	24.72
*Ptprz1*	protein tyrosine phosphatase, receptor Z, polypeptide 1	0.019	22.38
*Rlbp1*	retinaldehyde binding protein 1	0.022	19.09
*Lect1*	leukocyte cell derived chemotaxin 1	0.027	17.24
*Fbln5*	fibulin 5	0.010	16.47
*Fbln2*	fibulin 2	0.017	16.06
*Spon1*	spondin 1, (f-spondin) extracellular matrix protein	0.037	16.03
*Gja1*	gap junction protein, alpha 1	0.019	15.11
*Aldoc*	aldolase C, fructose-bisphosphate	0.017	14.26
*Pcdh10*	protocadherin 10	0.014	14.10
*Hes5*	hairy and enhancer of split 5 (Drosophila)	0.083	14.00
*Jam2*	junction adhesion molecule 2	0.031	13.82
*Tyrp1*	tyrosinase-related protein 1	0.019	13.57
*Tmem47*	transmembrane protein 47	0.023	12.97
*Cdh11*	cadherin 11	0.029	12.20
*Atp1a2*	ATPase, Na+/K+ transporting, alpha 2 polypeptide	0.017	12.08
*Megf10*	multiple EGF-like-domains 10	0.022	11.90
*Ttyh1*	tweety homolog 1 (Drosophila)	0.010	11.65
*Cybrd1*	cytochrome b reductase 1	0.042	11.55
*Ackr3*	atypical chemokine receptor 3	0.023	11.54
*Ptgfrn*	prostaglandin F2 receptor negative regulator	0.021	11.17
*Prss35*	protease, serine 35	0.021	11.17
*Lgr5*	leucine rich repeat G protein coupled receptor 5	0.022	11.15
*Gja1*	gap junction protein, alpha 1	0.021	10.97
*Atp1a2*	ATPase, Na+/K+ transporting, alpha 2 polypeptide	0.019	10.90
*Ndnf*	neuron-derived neurotrophic factor	0.025	10.80

(n = 3, two-way ANOVA with Benjamini-Hochberg correction. Selected genes, all p < 0.1 plus fold change >2)

### A distinct expression pattern for adjacent intact neurons

Gene regulation in damaged neurons compared to their intact neighbours shows the same trend as regulation compared to contralateral neurons, yet to a smaller degree (**Tables 4**and **5 and S3 Table**). In general, especially gene expression of adjacent neurons varied considerably between runs, as revealed by cluster analysis, supporting the hypothesis that adjacent neurons form a distinct but heterogeneous group of neurons in the DRG.

**Table 4:** Genes upregulated in damaged DRG neurons compared to adjacent non-damaged neurons.

*Symbol*	*Gene name*	*adjusted p*	*upregulation*
*Serpinb1a*	serine (or cysteine) peptidase inhibitor, clade B, member 1a	0.081	11.77
*Shisa9*	shisa homolog 9 (Xenopus laevis)	0.053	11.74
*Sox11*	SRY (sex determining region Y)-box 11	0.051	10.84
*Inhbb*	inhibin beta-B	0.098	8.52
*Gpr151*	G protein-coupled receptor 151	0.070	7.82
*Gna14*	guanine nucleotide binding protein, alpha 14	0.056	7.32
*Mmp16*	matrix metallopeptidase 16	0.056	7.23
*Gna14*	guanine nucleotide binding protein, alpha 14	0.056	5.90
*Lmo7*	LIM domain only 7	0.082	5.74
*Sez6l*	seizure related 6 homolog like	0.067	5.60
*Npy2r*	neuropeptide Y receptor Y2	0.087	5.10
*Sertm1*	serine rich and transmembrane domain containing 1	0.050	5.02
*Akap6*	A kinase (PRKA) anchor protein 6	0.083	4.69
*Slc6a19*	solute carrier family 6 (neurotransmitter transporter), member 19	0.091	4.67
*Thy1*	thymus cell antigen 1, theta	0.062	4.48
*Fgf3*	fibroblast growth factor 3	0.100	4.42
*Cacna2d1*	calcium channel, voltage-dependent, alpha2/delta subunit 1	0.057	4.37
*P2rx3*	purinergic receptor P2X, ligand-gated ion channel, 3	0.096	4.29
*Qrfpr*	pyroglutamylated RFamide peptide receptor	0.056	4.28
*Gap43*	growth associated protein 43	0.071	4.23
*Pcsk2*	proprotein convertase subtilisin/kexin type 2	0.062	4.06
*Esd*	esterase D/formylglutathione hydrolase	0.072	3.96
*Lynx1*	Ly6/neurotoxin 1	0.079	3.88
*Gnpnat1*	glucosamine-phosphate N-acetyltransferase 1	0.089	3.82
*St8sia1*	ST8 alpha-N-acetyl-neuraminide alpha-2,8-sialyltransferase 1	0.069	3.78
*Gal*	galanin	0.050	3.74
*Stmn2*	stathmin-like 2	0.056	3.58
*Pde7a*	phosphodiesterase 7A	0.093	3.36

(n = 3, two-way ANOVA with Benjamini-Hochberg correction. Selected genes, all p < 0.1 and fold change >2)

**Table 5:** Genes downregulated in damaged DRG neurons compared to adjacent non-damaged neurons.

*Symbol*	*Gene name*	*Adjusted p*	*downregulation*
*Aqp4*	aquaporin 4	0.057	18.34
*Bcan*	brevican	0.046	15.23
*Ptprz1*	protein tyrosine phosphatase, receptor type Z, polypeptide 1	0.053	14.11
*Fbln5*	fibulin 5	0.046	12.36
*Gja1*	gap junction protein, alpha 1	0.051	10.97
*Fbln2*	fibulin 2	0.051	10.02
*Tmem47*	transmembrane protein 47	0.060	9.62
*Tyrp1*	tyrosinase-related protein 1	0.055	9.22
*Adamts5*	a disintegrin-like and metallopeptidase (reprolysin type) with thrombospondin type 1 motif, 5 (aggrecanase-2)	0.062	9.08
*Aldoc*	aldolase C, fructose-bisphosphate	0.051	9.04
*Gja1*	gap junction protein, alpha 1	0.056	8.75
*Prss35*	protease, serine 35	0.056	8.59
*Hey2*	hairy/enhancer-of-split related with YRPW motif 2	0.070	8.30
*Atp1a2*	ATPase, Na+/K+ transporting, alpha 2 polypeptide	0.051	8.25
*Plscr2*	phospholipid scramblase 2	0.061	7.72
*Mlc1*	megalencephalic leukoencephalopathy with subcortical cysts 1 homolog (human)	0.062	7.64
*Slc35f1*	solute carrier family 35, member F1	0.065	7.59
*Cdh11*	cadherin 11	0.083	7.47
*Atp1a2*	ATPase, Na+/K+ transporting, alpha 2 polypeptide	0.051	7.37
*Slc7a2*	solute carrier family 7 (cationic amino acid transporter, y+ system), member 2	0.056	7.29
*Gpr37l1*	G protein-coupled receptor 37-like 1	0.051	7.20
*Lpar1*	lysophosphatidic acid receptor 1	0.050	7.14
*Cyr61*	cysteine rich protein 61	0.056	7.04
*Gja1*	gap junction protein, alpha 1	0.059	7.03
*Ttyh1*	tweety homolog 1 (Drosophila)	0.050	6.64
*Ntrk2*	neurotrophic tyrosine kinase, receptor, type 2	0.056	6.56
*Car2*	carbonic anhydrase 2	0.092	6.55
*Ptgfrn*	prostaglandin F2 receptor negative regulator	0.062	6.38
*Bcan*	brevican	0.062	6.21

(n = 3, two-way ANOVA with Benjamini-Hochberg correction. Selected genes, all p < 0.1 and fold change >2)

### Upregulation of CRH in DRG neurons in CCI

To validate microarray and qPCR results for *Crh*, we further analyzed protein expression of CRH by immunohistochemistry of L5 DRG. In DRG from sham animals, CRH immunoreactivity was very low and nearly undetectable. DRG from neuropathic animals, in contrast, showed a robust immunoreactivity of CRH, which was detected on the cytoplasm of small, medium and large size cell bodies (**Fig 6B**).

## Discussion

In this study, we examined the expression in primarily damaged DRG neurons compared to both adjacent intact neurons and corresponding contralateral neurons using fluorescent neuronal tracers. Using this strategy, we found that peripheral nerve injury induces selective changes in neuronal gene expression including genes linked with development of neuropathic pain. These findings provide us with an insight into the molecular changes in neuronal subpopulations in DRG in response to peripheral nerve injury. This neuron- and damage-specific approach better reflects previous findings (as reviewed by [2]) that emphasize different expression patterns in damaged and adjacent neurons as well as contributions of non-neuronal DRG cells to neuropathic features.

### Fluorescence neuron-specific labelling

The flow cytometric pattern for ipsilateral neurons consistently uncovered a subpopulation of DiI^-^/FE^+^ cells, suggesting strong neuronal damage. The large number of cells negative for both DiI and FE confirmed that a large percentage of DRG cells are of non-neuronal origin. In addition, other factors might play a role, like limited uptake of the fluorescent dye or afferent input from other areas. The percentage of intact neurons differed between FACS runs for microarray and qPCR, respectively, due to several reasons e.g. additional elimination for CD45^+^. Cluster analysis demonstrated a high consistency within damaged and contralateral neurons, respectively. In general, microarray results were validated for selected genes through qPCR. Therefore, our approach resulted in highly specific reproducible transcriptional profiles

In mRNA analysis, the number of genes differentially regulated compared to other conditions was by far the highest for damaged neurons. Interestingly, the difference was more pronounced in damaged neurons versus contralateral than versus adjacent neurons. This gives the latter an “intermediate” position thus possibly indicating a trickle-down effect. Such changes in neighboring tissue are in line with previous descriptions (e.g. reviewed by [2]). Alternatively, the DiI+/FE- group might contain affected cells with damage too subtle to allow FE uptake.

### Regulation patterns in damaged neurons are largely congruent with literature

A number of genes known to play a role in neuropathic pain have been differentially upregulated. Among them are several ion channels: ATP-sensing purinergic receptor P2rx3 has been long considered one of the major factors in neuronal sensitization, as has calcium channel subunit α2δ1, the target of gabapentin [21]. Channels down-regulated include various potassium channels (e.g. Kcnj10, Kcnn4). Voltage-gated potassium channel Kcnk2 (TREK-1) has been described as a polymodal pain sensor in small sensory neurons, regulated by GPCRs and co-localized with TRPV1. Neuropeptides differentially regulated include galanin and its receptor Gpr151. Their upregulation in damaged and, to lesser extent, in adjacent neurons is concordant with previous findings [22]. Other examples include neurotensin and cholecystokinin (CCK) which have been described mainly in nociceptive pain. Chemokine Ccl2 is an inflammatory and pain mediator released from primary afferents in the dorsal horn spinal cord [23]. It is co-localized with classical “neuropathic” peptides like substance P and CGRP and thought to potentiate glutaminergic receptors (AMPA/NMDA) as well as inhibit GABAergic receptors (GABA_A_) [24]. All three are downregulated in this study, as already described e.g. by Méchaly et al. [3]. The high congruence with literature data and the repeated reference to nociceptive/neuropathic pathways highly supports the selected approach.

### Novel regulated genes in neuropathic pain

Further genes that were regulated in the experiment have not been described in neuropathic pain before. Yet, their properties and known functions make a role in neuropathy plausible. In terms of neuronal damage, the upregulation of *Chac1*, part of an apoptotic pathway and downstream transcription factor *Atf3* [31], seems noteworthy. Several genes highly upregulated encode for proteins involved in axonal growth and neuronal differentiation, like syndecan1, AMPAR-associated Shisa9, Sox 11, or kainate-receptor modulator Neto1. Neto1 shapes both the biophysical properties and synaptic localization of glumatate receptors like NMDA receptor to modulate synaptic transmission [25]. Moreover, brevican, a chondroitin sulphate proteoglycan with growth-inhibiting features, was underexpressed in both damaged and adjacent neurons, as was aquaporin 4. This water channel has very recently been described as absent in degenerative (central) neurons [26]. Another group of upregulated genes hint at the inflammatory component of neuropathic pain, e.g. several CC chemokines, seizure-related gene 6, peptidase inhibitor serpinb1a, or annexin 1. Interestingly, *Ripk4*, a receptor-interacting serin-threonine kinase known to play a role in inflammatory cutaneous processes as well as B cell lymphoma [27], is highly downregulated. The fact that many of these hitherto unknown regulations are also found for adjacent neurons underlines the importance of not only a cell type-specific approach but also a differentiation of bystanders. This is particularly true for CRH.

### CRH as a possible player in neuropathic pain

CRH was upregulated in damaged neurons more than 200-fold compared to contralateral and 19-fold compared to adjacent neurons. The role of CRH and its receptors (CRH-R1 and CRH-R2) in neuropathy has not yet been well-defined. By now, two working mechanisms have been proposed: endogenous analgesia and nerve regeneration. In animal models, neuropathic pain can be alleviated by direct application of CRH to the nerve. As in inflammatory pain, this is caused by release of opioid peptides from infiltrating leukocytes. The analgesic effect can by antagonized by application of naloxone [28]. However, little has been found yet as to which cells express endogenous CRH in neuropathy. In the periphery, an increased expression of CRH and its receptors has mainly been shown in immune cells [29,30]. Moreover, a co-overexpression of CRH with pain-relevant neuropeptides has been observed in DRG and nociceptors [31]. Another hypothesis suggests a role in nerve regeneration by releasing brain-derived neurotrophic factor (BDNF) and promoting axonal outgrowth [13]. So far, neuronal CRH expression has been described mainly in the hypothalamus [32]: only little is known about its role in sensory neurons. Kim EH et al. [33] showed an increased immunoreactivity of CRH and its receptors in contralateral DRGs neurons after deafferential pain in rats. The differential neuron-specific approach of this experiment showed for the first time an upregulation in primarily damaged DRG neurons as well as, to a lesser extent, in their intact bystanders compared to contralateral DRG neurons. The results could be validated in immunohistochemistry staining of DRGs in neuropathic mice compared to sham controls. This suggests a central role for local neuronal CRH in neuropathic pain. To further elucidate its role, e.g. as analgesic agent or as promoter of axonal regeneration, a conditional knock-out approach in mice would be fruitful.

The example of CRH underlines the merits of the differential fluorescent tracing model presented here. Not only does this cell type-specific approach give a more detailed insight into gene regulation than a whole DRG screening. Moreover, a separate analysis of damaged and adjacent intact DRG neurons is crucial towards a more detailed understanding and respective functional characterization of both groups.

## Summary and Conclusion

mRNA expression profiles in damaged ipsilateral versus non-damaged contralateral DRG neurons of mice with neuropathic pain (CCI) revealed a specific profile partly present also in adjacent intact DRG neurons. Some regulated expressed genes confirmed results from previous studies; others are novel candidates deserving further investigation. CRH was the most prominent upregulated gene and was validated through qPCR and immunohistochemistry. Future studies should delineate the role of endogenous CRH in damaged neurons in more detail in different neuropathic pain models. In summary, the combination of differential fluorescent neuronal labelling with FACS described here is a promising approach for a more detailed understanding of transcriptional regulation in different neuronal subsets in neuropathic pain.

## Supporting Information

S1 Fig Flow cytometric detection of damaged and intact neurons for microarray. Seven days after CCI, DRGs L3-5 were excised and cells isolated. The sorting strategy to identify neurons positive for Fluoroemerald (FE) and DiI is shown in (**A**). Initially, cells were gated for size and granularity, before excluding dead cells using Sytox Blue. The remaining cells were sorted for DiI and FE. FACS plots of negative control (**B left**), contralateral (**B middle**) and ipsilateral (**B right**) DRG cells. DiI^+^/FE^-^ cells are considered to be spared neurons, FE^+^ cells are damaged neurons. Both populations were obtained for further analysis (n = 3, representative example).
(TIF)

S1 Table Primer sequences for qPCR. Primer sequences for 40 genes, including reference genes. Forward and reverse, 5’-3’.
(PDF)

S2 Table Genes regulated between contralateral and damaged neurons. Analysis of microarray results. Included are all genes with p < 0.1 and fold change >2. (n = 3, two-way ANOVA with Benjamini-Hochberg correction).
(PDF)

S3 Table Genes regulated between adjacent and damaged neurons. Analysis of microarray results. Included are all genes with p < 0.1 and fold change >2. (n = 3, two-way ANOVA with Benjamini-Hochberg correction).
(PDF)
