# Supplementary figures and images for: Differential Transcriptional Profiling of Damaged and Intact Adjacent Dorsal Root Ganglia Neurons in Neuropathic Pain
Source: PLoS One. 2015 Apr 16;10(4):e0123342. doi: 10.1371/journal.pone.0123342 (PMC4400143; doi:10.1371/journal.pone.0123342)

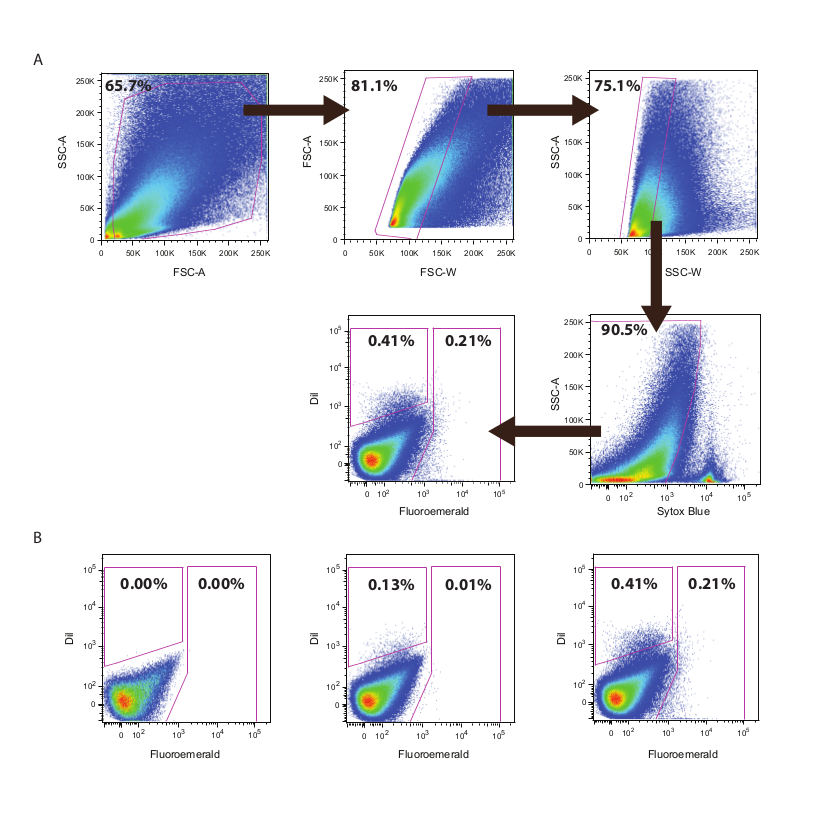

Supplement: S1 Fig — Seven days after CCI, DRGs L3-5 were excised and cells isolated. The sorting strategy to identify neurons positive for Fluoroemerald (FE) and DiI is shown in (A). Initially, cells were gated for size and granularity, before excluding dead cells using Sytox Blue. The remaining cells were sorted for DiI and FE. FACS plots of negative control (B left), contralateral (B middle) and ipsilateral (B right) DRG cells. DiI+/FE- cells are considered to be spared neurons, FE+ cells are damaged neurons. Both populations were obtained for further analysis (n = 3, representative example). (TIF) [file pone.0123342.s001.tif]
