# Supplementary material for: Differential Transcriptional Profiling of Damaged and Intact Adjacent Dorsal Root Ganglia Neurons in Neuropathic Pain
Source: PLoS One. 2015 Apr 16;10(4):e0123342. doi: 10.1371/journal.pone.0123342 (PMC4400143; doi:10.1371/journal.pone.0123342)
Supplement: S1 Table — Primer sequences for 40 genes, including reference genes. Forward and reverse, 5’-3’. (PDF) [file pone.0123342.s002.pdf]

Gene	*Forward primer 5'-3'*	*Reverse primer 5'-3'*
Npy	ATACTACTCCGCTCTGCGAC	GGGCTGGATCTCTTGCCATA
Tac1	GGACATGGCCAGATCTCTCA	TCTTTCGTAGTTCTGCATCGC
Gal	AGAAGAGAGGTTGGACCCTGA	GAGGCCATGCTTGTCGCTA
Cgrp	TTGTCAGCATCTTGCTCCTGT	TTCCAAGATTGACCTCAAAGGCA
Nts	AGCCCTGGAGGCAGATCTAT	AGGACTTGCTTTGCTGATCTTG
Cckbr	TCTCCCTCCTCAACAGCAGTA	CATAAAGGGTGATTCGAATGGTCA
Scn3a	AAAACCTCAGAAGCCCATCCC	TGTCTGGTTACAAAGTCAAAGACC
Scn10a	ATAAGGGGCCAGGACATCTTC	AAACACGAAGCCCTGGTACT
Trpa1	TGCAAAGAAGTGATCCAAATTTTCC	ACAAGGGCAACACGAAGATG
Trpv1	TTGCCCGGAAGACAGATAGC	AATGCTGTCTGGCCCTTGTA
Trpm3	GGTTAAGTTCCAAGCAGGGGT	GGTGCTCGGTATGATGTGAAC
Cacna2d	AGGCTGACTCCCAACTACAC	GTTGGTAGTCTGGGGCTTGA
P2rX3	TTGGGATCATCAACCGAGCC	AGAAAACCCACCCCACAAAGT
Kcnq2	CACTTTGAGAAACGGCGGAA	TGCGTGAGAGGTTAGTAGCAT
Chac1	CATAGGGGCAGCGACAAGAT	GCTCCCCTCGAACTTGGTA
Aqp4	CCAGGGAAGGCATGAGTGAC	CAGACTCCTTTGAAAGCCACC
Scn9a	GTGGGCGAATTCACCTTCCT	TCGAAGAGCTGAAACATTGCCTA
Shisa9	CAGGCAAAGAGCTCAACAAGT	GTGCCTTCTTTTGGCGTAGAA
Sox11	AGCGAGAAGATCCCGTTCAT	GGTCCGTCTTGGGCTTTTTG
Sdc1	GTCTGGGCAGCATGAGACG	TCAGGAGGAACATTTACAGCCA
Atf3	TGCGCTGGAGTCAGTTACC	CCGCCTCCTTTTCCTCTCATC
Serpinb1a	ATTGAGGACGAGTCCACGG	CCAAGTTCTCACGTTTGGTCC
Anxa1	TTGCCGAGAAGCTGTACGAA	CGAACGGGAGACCATAATCCT
Ngf	AGCTTTCTATACTGGCCGCA	GCCTGTACGCCGATCAAAAA
Crh(1)	GGGCTCACCTACCAAGGGA	CGACAGAGCCACCAGCA
Crh(2)	GTTAGCTCAGCAAGCTCACAG	GCCAAGCGCAACATTTCATTT
Otop1	GAATGAGCACAAGGAACGGC	TGTGGTGTGTGGTCATCCAA
Neto1	AGTCTATGGGATTTTCAGCTCGAT	TGGCAATGGTTTCAAAACTCCA
Mmp16	GCGGAACGGAGCAGTATTTC	TGACATTCTGGGGTCAGTCG
Ripk4	CGAATTCGTGAGAAGAGCCG	GCACACCCCAGATCACAATG
Gpr151	ACTGGAGGACCATCATTCCG	GAATGCCAATCACACACAGGT
Lmo7	CAGGCTCTCAGCTACGGAAT	ATGATCATGGCGGCTCCTT
Inhbb	AGATCATCAGCTTTGCAGAGACA	GACGAAGAAGTACAGGCGGA
Lect1	GAGACCTTTAAAATGGAAGCGG	TCTCCTCCAGCAAAACGGAT
Ccl2	GTTCACAGTTGCCGGCTG	TGGGATCATCTTGCTGGTGAA
Crhr1	GCTCTTCGCTCTGGGATGT	GTTCAGCCCCAGAAGGAGAA
Crhr2	AGCCCCTGTGGACACTTTTG	TCCAAGGTCGTGTTGCAGTA
Oprm1	CACCCCTCCACGGCTAATAC	TCCTGTAAAGATCTGGGCCAC
Oprd1	AACGTGCTCGTCATGTTTGG	TAGATGTTGGTGGCGGTCTT
Oprk1	GGTATTTGTGGTGGGCTTAGTG	TGCGGTCTTCATCTTCGTGTAT

UbC	GTGTTACCACCAAGAAGGTCAAA	ACTAAGACACCTCCCCCATCA
GAPDH	AAGGGCTCATGACCACAGTC	ATCACGCCACAGCTTTCCA
Advillin	GTACTTCACCTGCTGCTTTGC	GGCTGTCTTCCTGGAACTGA
