# Supplementary material for: Differential Transcriptional Profiling of Damaged and Intact Adjacent Dorsal Root Ganglia Neurons in Neuropathic Pain
Source: PLoS One. 2015 Apr 16;10(4):e0123342. doi: 10.1371/journal.pone.0123342 (PMC4400143; doi:10.1371/journal.pone.0123342)
Supplement: S2 Table — Analysis of microarray results. Included are all genes with p < 0.1 and fold change >2. (n = 3, two-way ANOVA with Benjamini-Hochberg correction). (PDF) [file pone.0123342.s003.pdf]

Gene	*probe ID*	*adjusted p*	*fold change*
Crh	1457984_at	0.064	207.68
Sprr1a	1449133_at	0.091	34.50
Inhbb	1426858_at	0.024	24.81
Neto1	1456283_at	0.081	19.93
Serpinb1a	1448301_s_at	0.033	17.05
Shisa9	1435424_x_at	0.020	14.18
Gpr151	1457555_at	0.023	14.15
Speer1-ps1	1452794_x_at	0.024	12.82
NA	1441815_at	0.024	12.61
Lmo7	1455056_at	0.023	11.82
NA	1439990_at	0.023	11.46
Cckbr	1454770_at	0.039	11.08
Sdc1	1415943_at	0.100	10.71
Sox11	1429372_at	0.022	10.70
Nts	1422860_at	0.070	10.63
Mmp16	1437568_at	0.050	10.60
NA	1431225_at	0.024	10.21
Serpinb1a	1416318_at	0.097	9.28
Sox11	1453125_at	0.022	9.26
Sox11	1453002_at	0.025	8.80
Chac1	1451382_at	0.062	8.52
Otop1	1438448_at	0.063	8.43
Sox11	1429051_s_at	0.037	8.37
P2rx3	1458396_at	0.025	8.20
Sez6l	1424764_at	0.024	7.89
Bcat1	1450871_a_at	0.093	7.43
Gal	1460668_at	0.010	7.22
Mmp16	1440161_at	0.024	6.89
Fgf3	1441914_x_at	0.035	6.86
Ecel1	1422586_at	0.044	6.75
Qrfpr	1457048_at	0.021	5.37
Sox11	1436790_a_at	0.060	5.21
Stmn4	1418105_at	0.048	4.88
Cers6	1434418_at	0.023	4.82
Flrt3	1429310_at	0.086	4.58
Spp1	1449254_at	0.026	4.45
Gpr31b	1452408_at	0.048	4.45
Slc6a19	1428595_at	0.065	4.39
Thy1	1423135_at	0.031	4.15
NA	1446324_at	0.040	4.12
Vgf	1436094_at	0.055	4.03
Cd109	1436346_at	0.024	3.99
Ano4	1442143_at	0.024	3.98

Arhgap19	1434911_s_at	0.056	3.96
Sdc1	1415944_at	0.078	3.95
Adam8	1416871_at	0.058	3.92
Mmp16	1422626_at	0.069	3.91
Hhipl1	1430062_at	0.023	3.91
Npy2r	1417489_at	0.085	3.86
Tes	1424246_a_at	0.022	3.85
Man1a	1417110_at	0.062	3.80
Onecut2	1460044_at	0.056	3.79
Mtus2	1434780_at	0.026	3.79
Prokr2	1437695_at	0.024	3.76
Cacna2d1	1425861_x_at	0.030	3.75
Akr1b8	1448894_at	0.042	3.71
Asns	1433966_x_at	0.062	3.70
Pappa2	1444451_at	0.048	3.65
Esd	1438488_at	0.048	3.63
Sertm1	1436444_at	0.021	3.61
Gap43	1423537_at	0.053	3.61
3632451O06Rik	1450770_at	0.052	3.60
Lgi2	1440147_at	0.071	3.59
Fgf3	1441350_at	0.065	3.53
Rgs20	1443694_at	0.046	3.37
Aim1	1426942_at	0.057	3.36
Stmn2	1423281_at	0.023	3.34
Ank3	1452872_at	0.100	3.29
Zwint	1427539_a_at	0.025	3.25
Sez6l	1440128_s_at	0.052	3.23
Tubb3	1415978_at	0.073	3.22
Xdh	1451006_at	0.083	3.21
Neto1	1425132_at	0.042	3.21
Hk2	1422612_at	0.037	3.20
Cacna2d1	1433643_at	0.023	3.19
Csprs	1435792_at	0.091	3.16
Il4ra	1421034_a_at	0.083	3.15
Tes	1460378_a_at	0.022	3.12
Tnik	1455256_at	0.032	3.07
Mbl2	1418787_at	0.073	3.06
Acsl1	1423883_at	0.047	3.02
P2rx3	1425093_at	0.066	2.99
Sel1l3	1452227_at	0.086	2.98
NA	1456593_at	0.042	2.97
Onecut2	1440005_at	0.044	2.91
Draxin	1456158_at	0.030	2.90
Gpr85	1437618_x_at	0.047	2.87

D430019H16Rik	1455447_at	0.081	2.86
Galnt9	1434055_at	0.031	2.82
Spsb1	1420150_at	0.077	2.81
Cacna2d1	1440397_at	0.027	2.80
Lppr4	1434685_at	0.067	2.80
Lce1f	1420550_at	0.084	2.78
Nsg1	1423055_at	0.050	2.74
Ina	1448991_a_at	0.077	2.74
Gpr85	1424896_at	0.020	2.74
Lynx1	1441952_x_at	0.097	2.72
2810037O22Rik	1428866_at	0.067	2.72
Gna14	1449848_at	0.050	2.71
Apba2	1427288_at	0.048	2.70
Chst1	1449147_at	0.022	2.69
Il13ra1	1427165_at	0.071	2.67
Tecta	1419632_at	0.026	2.66
Fam53b	1455428_at	0.098	2.66
Ank3	1447259_at	0.047	2.64
Lynx1	1417283_at	0.091	2.64
Slc6a19	1455442_at	0.035	2.63
1700025G04Rik	1455732_at	0.023	2.62
Slc41a2	1452445_at	0.065	2.62
Pde1c	1436251_at	0.058	2.60
Adam19	1418402_at	0.051	2.60
Hn1	1416028_a_at	0.062	2.57
Nkain1	1449553_at	0.024	2.57
Eif4ebp1	1434976_x_at	0.022	2.56
Smad1	1448208_at	0.019	2.55
Cyb5r1	1424048_a_at	0.023	2.55
Chst2	1422758_at	0.048	2.55
Cyp26a1	1419430_at	0.022	2.54
A830010M20Rik	1436117_at	0.022	2.53
Irak3	1435040_at	0.074	2.53
Stmn2	1423280_at	0.070	2.53
Vash2	1451105_at	0.049	2.51
Plxna4	1457840_at	0.095	2.51
Ppfia2	1456856_at	0.086	2.50
A630001G21Rik	1440214_at	0.085	2.50
Prokr2	1440564_at	0.067	2.50
Lppr1	1436733_at	0.050	2.49
Tgif1	1422286_a_at	0.096	2.45
Zwint	1429787_x_at	0.023	2.45
S100a11	1460351_at	0.094	2.45
Trf	1425546_a_at	0.085	2.44

Prr13	1423686_a_at	0.073	2.43
B4galnt4	1435913_at	0.028	2.42
A830039N20Rik	1455554_at	0.032	2.41
Olfml3	1448475_at	0.025	2.41
Arg2	1418847_at	0.084	2.40
Nrip1	1434384_at	0.092	2.39
Onecut2	1444980_at	0.024	2.39
1110046J04Rik	1457779_at	0.049	2.39
Spsb1	1449752_at	0.087	2.38
Rhoq	1427918_a_at	0.055	2.34
Il17ra	1420905_at	0.020	2.33
Sycp2	1444122_at	0.048	2.32
Liph	1457026_at	0.090	2.31
Tgfbi	1448123_s_at	0.081	2.31
Nup93	1424291_at	0.027	2.31
BB319198	1435598_at	0.091	2.30
Cpne5	1442166_at	0.027	2.28
Rasd2	1427344_s_at	0.052	2.28
Ackr1	1432273_a_at	0.082	2.28
Zbed4	1427090_at	0.022	2.28
NA	1460070_at	0.057	2.27
Cda	1427357_at	0.044	2.25
Lppr4	1427247_at	0.090	2.25
Aldh1l2	1436119_at	0.070	2.25
Rab33a	1417529_at	0.037	2.24
Nrip3	1448954_at	0.044	2.23
Spint1	1416627_at	0.098	2.21
Evl	1434920_a_at	0.091	2.20
Vasp	1451097_at	0.027	2.19
P2rx3	1431321_at	0.045	2.19
Myom1	1420693_at	0.066	2.19
Ppbp	1418480_at	0.024	2.19
Zbtb42	1456846_at	0.058	2.18
Rgs17	1439779_at	0.036	2.18
Asphd1	1456837_at	0.030	2.17
Setd4	1460373_a_at	0.041	2.17
Ugcg	1435133_at	0.052	2.17
Ptprn	1416588_at	0.070	2.17
Rundc3a	1449246_at	0.048	2.16
Amer3	1436407_at	0.084	2.15
Il16	1448686_at	0.094	2.15
Elovl7	1440354_at	0.058	2.14
Nrip1	1449089_at	0.055	2.14
Casp3	1449839_at	0.023	2.14

Zwint	1427540_at	0.070	2.14
Avil	1419148_at	0.094	2.13
Tubb2a-ps2	1419965_at	0.068	2.13
Cpd	1434547_at	0.062	2.12
Tgfbi	1456250_x_at	0.047	2.10
Plaur	1452521_a_at	0.021	2.10
Gpc1	1417389_at	0.100	2.09
Kpna3	1421828_at	0.022	2.09
Syt4	1415845_at	0.074	2.09
Gfer	1452272_a_at	0.023	2.08
Tbc1d9	1455015_at	0.037	2.08
Zbtb46	1429168_at	0.090	2.08
Dcx	1418139_at	0.090	2.07
NA	1441885_s_at	0.099	2.07
Rsad1	1437449_at	0.046	2.07
Plcl1	1445723_at	0.082	2.06
Shc2	1441356_at	0.054	2.06
C030004G16Rik	1432378_at	0.044	2.05
1700025G04Rik	1436431_at	0.093	2.05
Prkag2	1451140_s_at	0.033	2.04
Tubb2a	1427838_at	0.063	2.04
AA467197	1434046_at	0.022	2.04
Cds1	1456114_at	0.029	2.03
Col24a1	1453418_at	0.099	2.02
Ube2w	1426398_at	0.065	2.02
9030617O03Rik	1424226_at	0.058	2.02
Thsd7a	1456130_at	0.024	2.02
B830032F12	1435957_at	0.093	2.01
Gpr85	1424897_at	0.034	2.01
Mllt11	1416313_at	0.057	2.01
Pdlim1	1416554_at	0.050	2.00
Prodh	1417629_at	0.050	0.50
Scd2	1415822_at	0.030	0.50
Zeb1	1446127_at	0.065	0.50
Dars	1423800_at	0.063	0.50
Pm20d1	1438980_x_at	0.053	0.50
Rcn2	1422449_s_at	0.040	0.50
Cand2	1429622_at	0.033	0.50
Bola3	1433970_at	0.031	0.50
0610007P14Rik	1454161_s_at	0.092	0.49
Zbtb20	1439278_at	0.080	0.49
Cnn3	1455570_x_at	0.055	0.49
Gdpd2	1429076_a_at	0.047	0.49

NA	1449602_at	0.055	0.49
1810037I17Rik	1424365_at	0.025	0.49
NA	1446053_at	0.040	0.49
Bckdhb	1427153_at	0.044	0.49
Rhbdl1	1426094_at	0.035	0.49
Timp2	1433662_s_at	0.035	0.49
Gm14057	1430285_at	0.096	0.49
Pkd2	1417753_at	0.042	0.49
Smo	1427049_s_at	0.059	0.49
Vim	1438118_x_at	0.042	0.49
Selm	1424394_at	0.033	0.49
Hibadh	1435967_s_at	0.046	0.49
9130230N09Rik	1437980_at	0.094	0.49
Hsd17b10	1438391_x_at	0.100	0.49
Rab34	1416591_at	0.091	0.49
Ddhd1	1455321_at	0.047	0.49
Aph1b	1456500_at	0.099	0.49
Cyhr1	1451153_a_at	0.023	0.49
Syt11	1449264_at	0.059	0.49
Zcchc24	1428431_at	0.042	0.49
Dlgap1	1453027_at	0.087	0.49
Hexim1	1419359_at	0.100	0.48
Jam2	1431416_a_at	0.066	0.48
Aldh9a1	1437398_a_at	0.040	0.48
Bmp1	1426238_at	0.071	0.48
NA	1436672_at	0.073	0.48
Eci1	1418321_at	0.043	0.48
Mcm4	1416214_at	0.048	0.48
Srf	1418255_s_at	0.059	0.48
Ttc38	1437269_at	0.027	0.48
4931406C07Rik	1454067_a_at	0.039	0.48
Tmem237	1435661_at	0.091	0.48
Hnrnpd	1458273_at	0.074	0.48
Ctnna1	1448149_at	0.047	0.48
Olfml2b	1423915_at	0.070	0.48
Ntn5	1446220_at	0.077	0.48
Ifitm3	1423754_at	0.058	0.48
Kbtbd11	1430073_at	0.042	0.48
Tceal1	1424634_at	0.065	0.48
Acsl3	1428387_at	0.089	0.48
2610316D01Rik	1458702_at	0.058	0.48
Gas2l3	1455980_a_at	0.084	0.48
Gne	1455583_at	0.037	0.48
C130021I20Rik	1455416_at	0.080	0.47

Aldh2	1448143_at	0.069	0.47
Aasdhppt	1456625_at	0.096	0.47
Rom1	1448996_at	0.034	0.47
Fmnl2	1428579_at	0.098	0.47
Ednrb	1426314_at	0.056	0.47
Fgfr1	1436551_at	0.065	0.47
Slc25a15	1420967_at	0.089	0.47
Sgcb	1419667_at	0.086	0.47
Rasa3	1415850_at	0.073	0.47
Sorbs1	1428471_at	0.053	0.47
Bbs1	1437310_at	0.027	0.47
Tmem254b	1428738_a_at	0.049	0.47
Tln2	1435700_at	0.065	0.47
Fars2	1439406_x_at	0.029	0.47
Mt1	1422557_s_at	0.032	0.47
Ssbp2	1449815_a_at	0.027	0.47
Epdr1	1450380_at	0.098	0.47
Pnkd	1418746_at	0.044	0.47
Fars2	1431354_a_at	0.086	0.47
Gpd1	1416204_at	0.077	0.47
Ppfibp1	1452759_s_at	0.074	0.47
Hacl1	1449047_at	0.025	0.47
Mib1	1433853_at	0.039	0.47
Dad1	1454860_x_at	0.062	0.47
Zfyve21	1424669_at	0.055	0.47
Hsd17b12	1450011_at	0.022	0.47
Zdhhc2	1452654_at	0.084	0.47
Abhd14a	1425699_a_at	0.042	0.47
Pdgfc	1449351_s_at	0.096	0.46
Ctf1	1449093_at	0.021	0.46
Dlg3	1416918_at	0.040	0.46
Pygl	1417741_at	0.051	0.46
Bex4	1428209_at	0.055	0.46
Tmem205	1430421_a_at	0.025	0.46
Hibadh	1423780_at	0.032	0.46
Gpt2	1455007_s_at	0.086	0.46
Arhgap5	1450896_at	0.076	0.46
Celsr2	1435336_at	0.082	0.46
Tmem218	1417174_at	0.096	0.46
Gpc6	1419688_at	0.053	0.46
Laptm4b	1438365_x_at	0.099	0.46
Acadm	1415984_at	0.039	0.46
Ptprt	1450174_at	0.047	0.46
Gas2l3	1437244_at	0.085	0.46

E2f5	1460207_s_at	0.038	0.46
Socs3	1416576_at	0.090	0.46
Jmjd8	1424743_at	0.095	0.46
Naf1	1452192_at	0.063	0.46
Tbc1d10a	1448587_at	0.020	0.45
Csad	1427981_a_at	0.056	0.45
Egfl8	1447953_at	0.067	0.45
Tpmt	1438087_at	0.049	0.45
Megf9	1433968_a_at	0.091	0.45
Hsd17b11	1421011_at	0.021	0.45
Ddah2	1416457_at	0.022	0.45
Dcc	1440487_at	0.059	0.45
Rhbdf1	1424138_at	0.076	0.45
Ext1	1417730_at	0.070	0.45
Cipc	1424914_at	0.077	0.45
Tmem132a	1416845_at	0.085	0.45
Tspan5	1417179_at	0.099	0.45
Acvr2a	1451004_at	0.042	0.45
Gm20559	1455581_x_at	0.045	0.45
Sh3pxd2a	1428914_at	0.025	0.45
Dixdc1	1435207_at	0.099	0.44
Rbmx	1426863_at	0.042	0.44
Cacng5	1434785_at	0.082	0.44
Fdxr	1416806_at	0.088	0.44
Pcdhb16	1441994_at	0.032	0.44
Nmb	1419405_at	0.044	0.44
Ifit3	1449025_at	0.085	0.44
Kank4	1436425_at	0.081	0.44
Foxd3	1422210_at	0.027	0.44
Trio	1433745_at	0.070	0.44
Efemp2	1417018_at	0.055	0.44
Ttc23	1448902_at	0.089	0.44
Pebp1	1415950_a_at	0.063	0.44
Smarcd3	1418467_at	0.090	0.44
Cstf3	1443909_at	0.074	0.44
NA	1439496_at	0.099	0.44
Fbp1	1448470_at	0.023	0.44
Igfbp4	1437405_a_at	0.098	0.44
Ramp2	1418189_s_at	0.083	0.44
Lrig1	1434210_s_at	0.033	0.44
Hapln1	1438020_at	0.023	0.43
Echdc2	1425788_a_at	0.093	0.43
B9d1	1417596_at	0.044	0.43
Rassf10	1453201_at	0.035	0.43

Igfbp4	1437406_x_at	0.083	0.43
Aass	1423523_at	0.060	0.43
Dhrs4	1451559_a_at	0.037	0.43
Rassf8	1452283_at	0.063	0.43
Ccdc157	1455055_at	0.089	0.43
Omg	1418212_at	0.023	0.43
Dync2li1	1428446_at	0.064	0.43
Igfbp4	1423757_x_at	0.069	0.43
Sgms1	1426576_at	0.084	0.43
Caskin2	1450691_at	0.085	0.43
Ddhd1	1454070_a_at	0.065	0.43
Usp27x	1436004_at	0.028	0.43
H2afv	1436596_at	0.022	0.43
Nrarp	1417986_at	0.083	0.43
Dag1	1426778_at	0.060	0.43
Mif	1416335_at	0.022	0.42
Lrrc1	1452411_at	0.032	0.42
Pde4d	1435280_at	0.066	0.42
Hepacam	1434727_at	0.048	0.42
Hsd17b10	1448286_at	0.020	0.42
Mtcl1	1429053_at	0.029	0.42
Kcnj16	1435094_at	0.075	0.42
Lcat	1417043_at	0.023	0.42
Egr3	1436329_at	0.022	0.42
Cdh20	1450312_at	0.025	0.42
A530047J11Rik	1440884_s_at	0.058	0.42
Tmem64	1454709_at	0.023	0.42
Hoxb5os	1447886_at	0.090	0.42
E2f5	1417444_at	0.057	0.42
Fkbp9	1437687_x_at	0.059	0.42
Rxrg	1418782_at	0.070	0.42
NA	1443196_at	0.070	0.42
Sgce	1420688_a_at	0.063	0.41
Ppp1r15a	1448325_at	0.080	0.41
Cnn3	1436836_x_at	0.048	0.41
Vamp5	1430522_a_at	0.023	0.41
Foxo1	1416982_at	0.093	0.41
Cipc	1424915_s_at	0.050	0.41
Mtfp1	1424223_at	0.024	0.41
Lrig3	1430554_at	0.100	0.41
Tcf7l2	1429428_at	0.075	0.41
Grk5	1449514_at	0.062	0.41
Prdm16	1429308_at	0.086	0.41
Egln3	1418649_at	0.024	0.41

Laptm4b	1436915_x_at	0.030	0.41
Vim	1456292_a_at	0.062	0.41
Ppp2r3a	1437869_at	0.034	0.41
Trim2	1417027_at	0.100	0.41
Pank3	1426259_at	0.059	0.40
Pon2	1429019_s_at	0.029	0.40
NA	1439322_at	0.058	0.40
Zbtb20	1437598_at	0.097	0.40
Acyp2	1427943_at	0.023	0.40
Fam101b	1456603_at	0.097	0.40
Il17rd	1429893_at	0.024	0.40
Tceal3	1450961_a_at	0.019	0.40
Col19a1	1456953_at	0.060	0.40
Bsg	1456616_a_at	0.021	0.40
Btd	1417987_at	0.063	0.40
9530077C05Rik	1452963_at	0.084	0.40
Tm7sf3	1452664_a_at	0.030	0.40
Tmem56	1434553_at	0.046	0.40
Cadm1	1417377_at	0.089	0.40
Clec14a	1419467_at	0.094	0.40
Igf1r	1428967_at	0.036	0.40
Rab38	1417700_at	0.031	0.40
Negr1	1456392_at	0.070	0.40
Ncald	1417568_at	0.039	0.40
Fnta	1447904_s_at	0.041	0.40
Dbp	1418174_at	0.038	0.40
Edil3	1433474_at	0.024	0.40
Gstm5	1416842_at	0.022	0.40
Fkbp9	1423677_at	0.067	0.40
Dad1	1418528_a_at	0.024	0.40
Cetn2	1418579_at	0.070	0.40
Ier2	1416442_at	0.023	0.40
Tmem204	1434621_at	0.073	0.39
Abhd6	1419103_a_at	0.077	0.39
NA	1443230_at	0.056	0.39
Arvcf	1423061_at	0.046	0.39
Tmem41b	1428586_at	0.023	0.39
Csmd1	1445531_at	0.067	0.39
Spon1	1442613_at	0.096	0.39
Ceacam1	1450494_x_at	0.062	0.39
Igsf3	1455049_at	0.099	0.39
Calca	1452004_at	0.040	0.39
Sh3pxd2b	1435644_at	0.030	0.39
Gja1	1438973_x_at	0.056	0.39

Acss2	1422479_at	0.059	0.39
Chdh	1455435_s_at	0.059	0.39
Nr2f2	1416160_at	0.088	0.39
Bmpr1a	1425492_at	0.064	0.39
Tril	1452961_at	0.090	0.39
Cdk6	1435338_at	0.023	0.39
Foxo1	1416983_s_at	0.062	0.39
NA	1439651_at	0.100	0.39
Fam213a	1447774_x_at	0.085	0.39
Chpt1	1435446_a_at	0.022	0.39
Rgcc	1438511_a_at	0.031	0.39
Acaa2	1455061_a_at	0.042	0.39
Idi1	1423804_a_at	0.044	0.39
Cdc42ep2	1428750_at	0.090	0.39
Syngr1	1419289_a_at	0.056	0.39
Pfkm	1416780_at	0.022	0.39
Kctd1	1425104_at	0.078	0.38
Pon2	1450686_at	0.046	0.38
Fam107a	1434203_at	0.089	0.38
Trip6	1449041_a_at	0.073	0.38
Mapre2	1426245_s_at	0.040	0.38
BC023202	1456908_at	0.071	0.38
Fam213a	1452716_at	0.034	0.38
Lrig1	1449893_a_at	0.082	0.38
Cldn12	1433782_at	0.086	0.38
Slc1a2	1438194_at	0.047	0.38
Abcg1	1455221_at	0.024	0.38
Tulp3	1449008_at	0.066	0.38
Nme7	1418217_at	0.023	0.38
Oat	1416452_at	0.022	0.38
Tox	1425484_at	0.023	0.38
Tmem64	1433735_a_at	0.022	0.38
Dag1	1423872_a_at	0.056	0.38
Tspan7	1448737_at	0.046	0.38
Epas1	1449888_at	0.070	0.38
Hrsp12	1428326_s_at	0.038	0.38
Lpar3	1418723_at	0.042	0.38
Nek8	1450337_a_at	0.042	0.38
Mapre2	1451990_at	0.090	0.38
Mapre2	1426244_at	0.046	0.38
Dag1	1426779_x_at	0.038	0.37
Fam117a	1433639_at	0.023	0.37
Ugt1a6a	1426261_s_at	0.091	0.37
Eci2	1431012_a_at	0.023	0.37

Kazald1	1436528_at	0.084	0.37
NA	1445965_at	0.082	0.37
Il17rc	1419671_a_at	0.081	0.37
Gsap	1427515_at	0.020	0.37
Nid2	1423516_a_at	0.062	0.37
Cntn1	1449563_at	0.048	0.37
Evi5	1417513_at	0.094	0.37
Pmepa1	1452295_at	0.071	0.37
Sqle	1415993_at	0.073	0.37
Fntb	1434309_at	0.044	0.37
2810468N07Rik	1452980_at	0.025	0.37
NA	1445894_at	0.042	0.37
Gm20559	1436172_at	0.090	0.37
Kcns2	1457325_at	0.060	0.37
Zswim5	1438224_at	0.056	0.37
Fam19a5	1419490_at	0.090	0.36
Nxn	1422466_at	0.080	0.36
Paqr8	1428958_at	0.075	0.36
Ephx1	1422438_at	0.035	0.36
Gamt	1422558_at	0.021	0.36
Lpar1	1426110_a_at	0.022	0.36
Dhh	1422127_at	0.066	0.36
Prickle2	1428808_at	0.090	0.36
NA	1420136_a_at	0.076	0.36
Rpl41	1454639_x_at	0.058	0.36
Mmd2	1444572_at	0.062	0.36
Fermt2	1434180_at	0.073	0.36
Laptm4b	1416148_at	0.023	0.36
4933431E20Rik	1434497_at	0.076	0.36
Pde12	1454963_at	0.056	0.36
Zfp383	1453212_at	0.067	0.36
Dbi	1455976_x_at	0.025	0.36
Retsat	1424716_at	0.030	0.36
Enpp5	1425702_a_at	0.036	0.36
NA	1455418_at	0.026	0.36
Fads1	1423680_at	0.022	0.36
Dkk3	1417312_at	0.070	0.36
Ece1	1455741_a_at	0.094	0.35
Foxo1	1416981_at	0.073	0.35
Plp1	1425468_at	0.043	0.35
Bcl2	1457687_at	0.021	0.35
Sox6	1434918_at	0.100	0.35
Grid2	1421436_at	0.066	0.35
Parva	1431375_s_at	0.076	0.35

Abhd4	1439259_x_at	0.033	0.35
Gria4	1435722_at	0.036	0.35
Cthrc1	1452968_at	0.056	0.35
Hsd17b11	1434642_at	0.026	0.35
Card10	1449491_at	0.078	0.35
Sc5d	1451457_at	0.092	0.35
Tmem64	1434307_at	0.040	0.35
Gm715	1445503_at	0.041	0.35
Daam2	1430247_at	0.026	0.35
Nsdhl	1416222_at	0.055	0.35
Plxnb3	1418750_at	0.019	0.35
Upp1	1448562_at	0.096	0.35
Tet1	1455425_at	0.031	0.35
Btbd3	1425660_at	0.039	0.34
Itga1	1455251_at	0.053	0.34
Insc	1453259_at	0.027	0.34
S100b	1434342_at	0.073	0.34
Tmprss5	1418959_at	0.079	0.34
Gcc1	1429033_at	0.058	0.34
AW011738	1436080_at	0.058	0.34
Gprc5c	1452947_at	0.060	0.34
Rab38	1439628_x_at	0.048	0.34
Sox10	1451689_a_at	0.099	0.34
Chil1	1451537_at	0.022	0.34
Tpd52l1	1418412_at	0.023	0.34
Zbtb20	1422064_a_at	0.045	0.34
Tmod2	1430153_at	0.064	0.34
Fam114a1	1441904_x_at	0.079	0.34
Rnf215	1452756_at	0.021	0.34
Cnn3	1456380_x_at	0.031	0.34
Usp54	1428731_at	0.022	0.34
Lgalsl	1451313_a_at	0.080	0.34
Ctnnal1	1420930_s_at	0.025	0.34
Slc24a3	1424308_at	0.047	0.34
Fjx1	1450728_at	0.038	0.34
Cyp51	1422534_at	0.099	0.33
Sort1	1450955_s_at	0.022	0.33
Dclk1	1424270_at	0.085	0.33
Sfrp1	1428136_at	0.086	0.33
Gas1	1416855_at	0.023	0.33
Pcdh10	1442565_at	0.025	0.33
Ldhb	1434499_a_at	0.047	0.33
Ehd2	1459823_at	0.096	0.33
Idi1	1451122_at	0.062	0.33

Rap1gap	1428443_a_at	0.022	0.33
Lcmt2	1433518_at	0.039	0.33
Stard4	1455011_at	0.030	0.33
Sgms1	1436499_at	0.030	0.33
Pex5l	1438399_at	0.046	0.33
Camk2d	1460630_at	0.065	0.33
Jam3	1423503_at	0.085	0.33
Asrgl1	1424396_a_at	0.087	0.33
Plscr1	1453181_x_at	0.037	0.33
Smoc1	1448321_at	0.051	0.33
Plscr1	1429527_a_at	0.022	0.33
Gpr125	1426782_at	0.023	0.33
Btbd3	1433868_at	0.040	0.33
Zfp191	1426895_at	0.023	0.33
Chrna7	1440681_at	0.066	0.33
Tmc7	1456981_at	0.091	0.33
Elovl6	1417404_at	0.028	0.33
Klhl13	1416242_at	0.025	0.33
Lhcgr	1450192_at	0.031	0.33
Tgfbr3	1433795_at	0.060	0.33
Gprasp2	1434073_at	0.078	0.32
Nfia	1456087_at	0.077	0.32
Gramd1b	1435402_at	0.036	0.32
Lrrc16a	1451804_a_at	0.019	0.32
Kif1b	1455182_at	0.057	0.32
Matn2	1455978_a_at	0.058	0.32
Prnp	1416130_at	0.049	0.32
Dhcr24	1418129_at	0.070	0.32
Afap1l2	1455833_at	0.032	0.32
Lurap1l	1428384_at	0.047	0.32
1190002N15Rik	1433582_at	0.022	0.32
Gm2115	1442595_at	0.021	0.32
Lsamp	1435895_at	0.085	0.32
Usp53	1444507_at	0.043	0.32
Micall2	1434322_at	0.055	0.32
Slc12a4	1417446_at	0.044	0.32
Plp1	1451718_at	0.029	0.32
Nav3	1456144_at	0.047	0.32
Echdc1	1419552_at	0.020	0.32
Tac1	1416783_at	0.085	0.32
NA	1436772_at	0.058	0.32
Itpr3	1417297_at	0.066	0.32
Igfbp4	1423756_s_at	0.039	0.32
Ptprt	1439725_at	0.036	0.32

6230424C14Rik	1453188_at	0.070	0.32
Mxi1	1450376_at	0.025	0.32
Smpd2	1416999_at	0.030	0.32
Efnb1	1418285_at	0.029	0.32
Bcl2	1437122_at	0.043	0.32
Adamts15	1427056_at	0.040	0.32
Hmgn3	1434875_a_at	0.049	0.32
Plxnb3	1440813_s_at	0.029	0.32
Chadl	1440958_at	0.022	0.31
Gm13111	1457215_at	0.063	0.31
Irf1	1448436_a_at	0.037	0.31
Ppp2r3a	1455091_at	0.027	0.31
Pcdh9	1442659_at	0.062	0.31
Plcd4	1437030_at	0.099	0.31
Smpdl3a	1416635_at	0.023	0.31
Dag1	1456131_x_at	0.050	0.31
Gpr37	1450875_at	0.057	0.31
Cd34	1416072_at	0.077	0.31
Chchd10	1436990_s_at	0.029	0.31
Ldhb	1455235_x_at	0.022	0.31
Slain1	1424824_at	0.025	0.31
Tnxb	1450798_at	0.032	0.31
Scd1	1415964_at	0.024	0.31
Fam171a1	1438402_at	0.039	0.31
Hsdl2	1426856_at	0.035	0.31
Dlgap1	1436076_at	0.070	0.31
Scarb1	1437378_x_at	0.093	0.31
Lphn3	1460440_at	0.031	0.31
AI852580	1459723_at	0.062	0.31
Proca1	1442508_at	0.027	0.31
Ppara	1449051_at	0.038	0.31
Mt2	1428942_at	0.094	0.31
Casp12	1418981_at	0.048	0.31
Gca	1451451_at	0.021	0.31
Scd1	1415965_at	0.062	0.31
40057.00	1417038_at	0.042	0.31
Lama4	1424808_at	0.098	0.31
Pde4b	1422473_at	0.031	0.30
Vegfa	1420909_at	0.048	0.30
Tmem229a	1444135_at	0.078	0.30
Ift122	1441259_s_at	0.029	0.30
Lrrtm2	1437787_at	0.043	0.30
Ddit4	1428306_at	0.084	0.30
Scgb1a1	1452543_a_at	0.099	0.30

Gucy1a2	1429579_at	0.056	0.30
Cacng4	1450975_at	0.058	0.30
Btbd11	1459838_s_at	0.021	0.30
Slc4a4	1421225_a_at	0.021	0.30
Acvr2a	1437382_at	0.042	0.30
Lrrn3	1434539_at	0.029	0.30
Gramd1b	1435229_at	0.025	0.30
Pgf	1418471_at	0.024	0.30
Sorcs1	1436662_at	0.047	0.30
Tfap2a	1421996_at	0.049	0.30
NA	1442823_at	0.090	0.30
Plekhf1	1424671_at	0.077	0.30
Fkbp10	1415951_at	0.062	0.30
Metrn	1427101_at	0.059	0.30
Trib2	1426640_s_at	0.044	0.30
Pvrl3	1423331_a_at	0.092	0.30
Bhlhe40	1418025_at	0.080	0.30
2010004M13Rik	1455646_at	0.049	0.30
Ceacam1	1427630_x_at	0.076	0.30
Ldhb	1416183_a_at	0.023	0.30
Plp1	1425467_a_at	0.023	0.30
Synpo2	1452879_at	0.099	0.30
Spry4	1445669_at	0.026	0.30
Lrrc4	1416097_at	0.032	0.30
Dbi	1438093_x_at	0.019	0.30
Adamts7	1452339_at	0.054	0.30
Slc1a5	1416629_at	0.086	0.29
Slc26a7	1436279_at	0.091	0.29
Greb1	1439568_at	0.043	0.29
Cdh15	1418602_at	0.035	0.29
Wwtr1	1437155_a_at	0.090	0.29
Adamts9	1437785_at	0.043	0.29
Nedd4	1421955_a_at	0.073	0.29
Lrp4	1426288_at	0.068	0.29
Sc5d	1434520_at	0.040	0.29
Pla2g16	1451611_at	0.022	0.29
Rhobtb3	1433647_s_at	0.024	0.29
Gm14005	1444524_at	0.058	0.29
Acaa2	1428145_at	0.056	0.29
Fdps	1423418_at	0.022	0.29
Elovl5	1437211_x_at	0.032	0.29
Ar	1455647_at	0.082	0.29
Fnta	1417465_at	0.021	0.29
Adhfe1	1424393_s_at	0.028	0.29

Gbp6	1438676_at	0.060	0.29
Erbb3	1434606_at	0.092	0.29
Epha7	1451991_at	0.020	0.29
Gpr56	1433485_x_at	0.090	0.29
Id1	1425895_a_at	0.051	0.29
Dbi	1433991_x_at	0.019	0.29
Sv2b	1435687_at	0.071	0.29
Pde4b	1422474_at	0.068	0.29
Rpl41	1433530_at	0.040	0.29
Alpl	1423611_at	0.027	0.29
Hs3st1	1423450_a_at	0.060	0.28
Grb14	1417673_at	0.042	0.28
Scarb1	1416050_a_at	0.020	0.28
Pcsk5	1437339_s_at	0.047	0.28
Add3	1423297_at	0.024	0.28
Slc7a2	1450703_at	0.058	0.28
Pcdh7	1456214_at	0.089	0.28
Sorbs1	1425826_a_at	0.067	0.28
Pla2g16	1445597_s_at	0.023	0.28
Slc22a17	1448209_a_at	0.029	0.28
Chpt1	1455901_at	0.029	0.28
Phlda2	1417837_at	0.017	0.28
Chpt1	1435870_at	0.054	0.28
Ppargc1a	1437751_at	0.057	0.28
Vim	1450641_at	0.029	0.28
Zfp191	1430651_s_at	0.096	0.28
Irs1	1423104_at	0.047	0.28
Tm7sf2	1460684_at	0.040	0.28
Lysmd2	1428626_at	0.025	0.28
Aif1l	1451287_s_at	0.090	0.28
Ssbp2	1438423_at	0.099	0.28
Ttyh1	1426617_a_at	0.022	0.28
Slco3a1	1448918_at	0.058	0.28
Notch1	1418634_at	0.041	0.28
Amot	1454890_at	0.036	0.28
Msmo1	1459627_at	0.084	0.27
Cyp51	1422533_at	0.053	0.27
Ldhb	1448237_x_at	0.024	0.27
Itih5	1429159_at	0.064	0.27
Mest	1423294_at	0.023	0.27
Col23a1	1429209_at	0.040	0.27
Dpysl4	1418298_s_at	0.066	0.27
Spon1	1424415_s_at	0.031	0.27
Sparc	1416589_at	0.037	0.27

Slc27a1	1435658_at	0.026	0.27
Fat1	1433857_at	0.070	0.27
Aldh1a7	1418601_at	0.053	0.27
Lama4	1424807_at	0.055	0.27
Ripk4	1418487_at	0.019	0.27
Cmtm5	1429798_s_at	0.023	0.27
Hpdl	1455120_at	0.038	0.27
Rcn1	1417090_at	0.062	0.27
Gabbr1	1437188_at	0.050	0.27
Psph	1415673_at	0.029	0.27
Hepacam	1429023_at	0.022	0.27
Nr2f2	1436475_at	0.042	0.27
Ppara	1439675_at	0.029	0.27
S100a16	1425560_a_at	0.049	0.27
Itgb8	1436223_at	0.026	0.26
Fmo2	1435459_at	0.065	0.26
Pcdh9	1429861_at	0.059	0.26
Hmgn3	1431777_a_at	0.024	0.26
Agt	1423396_at	0.027	0.26
Pdpn	1419309_at	0.029	0.26
Mpzl1	1428167_a_at	0.042	0.26
Hmgcr	1427229_at	0.026	0.26
Spry2	1436584_at	0.022	0.26
Synpo	1434089_at	0.028	0.26
Zfp503	1423835_at	0.022	0.26
Dhh	1434959_at	0.022	0.26
Enpp1	1459546_s_at	0.047	0.26
A030001O10Rik	1434989_at	0.024	0.26
Col4a1	1426348_at	0.044	0.26
NA	1457373_at	0.024	0.26
Ifit1	1450783_at	0.047	0.26
Tspan6	1448501_at	0.063	0.26
Lss	1420013_s_at	0.022	0.26
Sv2b	1434800_at	0.077	0.26
Olfml2a	1455743_at	0.048	0.25
Dpyd	1427945_at	0.047	0.25
Abhd3	1417946_at	0.022	0.25
Slc12a2	1417622_at	0.020	0.25
Fzd3	1434788_at	0.025	0.25
Fam198b	1416805_at	0.070	0.25
Me1	1430307_a_at	0.042	0.25
Msmo1	1423078_a_at	0.042	0.25
Gpm6a	1456741_s_at	0.097	0.25
Dusp4	1428834_at	0.055	0.25

Kcnk2	1449158_at	0.042	0.25
Afap1l2	1436870_s_at	0.047	0.25
Add3	1423298_at	0.041	0.25
Dkk3	1448669_at	0.042	0.25
Dennd2a	1433500_at	0.058	0.25
Pcsk5	1451406_a_at	0.046	0.25
Fxyd1	1421374_a_at	0.084	0.25
Emp2	1433670_at	0.042	0.25
Pnmal2	1435960_at	0.018	0.25
Aldh1l1	1424400_a_at	0.014	0.25
Dmd	1417307_at	0.062	0.25
Dmd	1448665_at	0.038	0.25
Pcdh10	1425563_s_at	0.033	0.25
Gpc6	1437417_s_at	0.050	0.25
Ceacam1	1425538_x_at	0.100	0.24
Rnd2	1422670_at	0.023	0.24
Trim2	1417028_a_at	0.047	0.24
Atp1a2	1443823_s_at	0.026	0.24
Tmem255a	1439006_x_at	0.099	0.24
Mpzl1	1428168_at	0.031	0.24
Fam19a5	1419489_at	0.044	0.24
Nlgn3	1436135_at	0.028	0.24
Ramp2	1418187_at	0.053	0.24
Grik3	1440177_at	0.082	0.24
Farp2	1440799_s_at	0.086	0.24
Dbi	1422432_at	0.023	0.24
Gpr56	1421118_a_at	0.057	0.24
NA	1445539_at	0.044	0.24
Ncam1	1450437_a_at	0.029	0.24
Shisa2	1423852_at	0.085	0.24
Lgi4	1434121_at	0.025	0.24
Abca8b	1427546_at	0.035	0.24
Usp53	1452385_at	0.022	0.24
Gpm6b	1423091_a_at	0.019	0.24
1190002N15Rik	1433581_at	0.023	0.24
Me1	1416632_at	0.035	0.24
Ift122	1427239_at	0.088	0.24
Elovl6	1417403_at	0.028	0.24
Acaa2	1428146_s_at	0.022	0.24
Grid1	1441499_at	0.042	0.23
Tmem255a	1436948_a_at	0.067	0.23
Col23a1	1429210_at	0.079	0.23
Fam107a	1434202_a_at	0.040	0.23
Tek	1418788_at	0.050	0.23

Sulf1	1438200_at	0.084	0.23
Phgdh	1426657_s_at	0.080	0.23
Tc2n	1443933_at	0.023	0.23
Tspan6	1416872_at	0.042	0.23
Fam198b	1429637_at	0.041	0.23
Srpx	1451939_a_at	0.042	0.23
Fzd2	1418533_s_at	0.090	0.23
Elovl5	1415840_at	0.027	0.23
Ptprg	1456070_at	0.047	0.23
Ssbp2	1451542_at	0.081	0.23
Ezr	1450850_at	0.089	0.23
Asrgl1	1424395_at	0.048	0.23
Ppap2b	1429514_at	0.040	0.23
Fam114a1	1417272_at	0.047	0.23
Frmd6	1451264_at	0.022	0.23
Fbxl7	1456220_at	0.021	0.23
Rarb	1454906_at	0.042	0.23
Trim2	1448551_a_at	0.037	0.23
Abca8a	1427371_at	0.028	0.23
Rbm46	1439944_at	0.027	0.23
Gldc	1416049_at	0.055	0.23
Gpr124	1418379_s_at	0.077	0.23
Dpyd	1427946_s_at	0.023	0.22
Lphn3	1428523_at	0.056	0.22
Kirrel	1434797_at	0.031	0.22
Gstt1	1418186_at	0.062	0.22
Myo16	1445532_at	0.023	0.22
Pard6g	1420851_at	0.048	0.22
Slitrk1	1428089_at	0.084	0.22
Atp1b2	1435148_at	0.023	0.22
Stard4	1429240_at	0.037	0.22
Dnah7b	1438466_at	0.093	0.22
Tgfb2	1423250_a_at	0.052	0.22
Trpm3	1439026_at	0.062	0.22
B130021B11Rik	1437558_at	0.042	0.22
C1qtnf5	1424762_at	0.025	0.22
Adamts9	1430352_at	0.082	0.22
Gpam	1419499_at	0.019	0.22
Gbp6	1447927_at	0.027	0.22
Tenm3	1429178_at	0.028	0.22
Ppargc1a	1434100_x_at	0.022	0.22
Entpd2	1418259_a_at	0.061	0.22
Tmem229a	1455963_at	0.079	0.21
NA	1457741_at	0.033	0.21

Tmod2	1431326_a_at	0.023	0.21
Lrrc4	1435832_at	0.031	0.21
Add3	1426574_a_at	0.024	0.21
Gpr126	1437408_at	0.022	0.21
Shisa2	1423851_a_at	0.037	0.21
Ppargc1a	1434099_at	0.027	0.21
Vwa1	1426399_at	0.067	0.21
Mgst1	1415897_a_at	0.024	0.21
Gca	1424698_s_at	0.077	0.21
Car5b	1447928_at	0.051	0.21
Kit	1452514_a_at	0.023	0.21
Prdm5	1432057_a_at	0.038	0.21
Col12a1	1434411_at	0.047	0.21
Fign	1442873_at	0.091	0.21
Slc7a2	1426008_a_at	0.037	0.21
Pcx	AFFX-PyruCarbMur/L09192_3_at	0.023	0.21
B130021B11Rik	1438989_s_at	0.028	0.21
Sfrp5	1423023_at	0.022	0.21
Sphk1	1451596_a_at	0.087	0.21
Vldlr	1417900_a_at	0.084	0.21
Mdk	1416006_at	0.024	0.21
Htra1	1416749_at	0.037	0.21
Ptpn13	1452127_a_at	0.033	0.21
Fam102a	1426894_s_at	0.035	0.21
NA	1445062_at	0.087	0.21
Fzd8	1423348_at	0.031	0.21
Cebpd	1423233_at	0.025	0.21
Serpine2	1416666_at	0.023	0.21
NA	1459497_at	0.054	0.21
Eepd1	1417877_at	0.019	0.20
Sorcs2	1419358_at	0.032	0.20
Ppp1r14c	1417701_at	0.070	0.20
Slco3a1	1434537_at	0.029	0.20
Frem2	1457038_at	0.025	0.20
Htra1	1438251_x_at	0.040	0.20
Enpp1	1419276_at	0.023	0.20
Pdzd2	1435553_at	0.056	0.20
Stard4	1429239_a_at	0.077	0.20
Gm2136	1457494_at	0.019	0.20
Phlpp1	1426994_at	0.059	0.20
Cdh1	1448261_at	0.022	0.20
Nr2f2	1416159_at	0.029	0.20
Jag1	1434070_at	0.056	0.20

Vldlr	1435893_at	0.084	0.20
Fads2	1443838_x_at	0.037	0.20
Plce1	1452398_at	0.079	0.20
Irgm1	1418825_at	0.051	0.20
Vldlr	1438258_at	0.064	0.20
Dnaic2	1436296_x_at	0.073	0.20
NA	1445268_at	0.023	0.20
Matn2	1419442_at	0.054	0.20
Abhd4	1416315_at	0.021	0.20
Fkbp10	1449632_s_at	0.061	0.20
Ppargc1a	1456395_at	0.024	0.19
NA	1455298_at	0.023	0.19
Car13	1421307_at	0.021	0.19
Adamts5	1450658_at	0.029	0.19
Vstm4	1455050_at	0.083	0.19
Gpsm2	1424895_at	0.093	0.19
Tmem40	1441917_s_at	0.065	0.19
Gstk1	1452823_at	0.023	0.19
Apcdd1	1449070_x_at	0.084	0.19
Fam181b	1417862_at	0.096	0.19
NA	1437442_at	0.053	0.19
Sox2	1416967_at	0.021	0.19
Car8	1427482_a_at	0.093	0.19
Pnmal2	1438363_at	0.087	0.19
Fads2	1449325_at	0.021	0.19
Car2	1448752_at	0.081	0.19
Nptx1	1434877_at	0.093	0.19
Ncam2	1421592_at	0.058	0.19
Arhgef26	1429185_at	0.022	0.19
Crym	1416776_at	0.019	0.19
Kcnj10	1419601_at	0.030	0.19
Cyp2d22	1419039_at	0.022	0.19
Pxdn	1428259_at	0.055	0.19
Pcx	1416383_a_at	0.021	0.19
Sostdc1	1449340_at	0.021	0.19
Slc4a4	1452071_at	0.019	0.18
Rarres2	1437902_s_at	0.049	0.18
Col4a4	1445328_at	0.073	0.18
Plscr4	1433626_at	0.032	0.18
A830082K12Rik	1458470_at	0.081	0.18
Ctnnd2	1456116_at	0.022	0.18
Prex2	1436569_at	0.021	0.18
S1pr3	1438658_a_at	0.022	0.18
Kctd1	1422293_a_at	0.052	0.18

Ndrg2	1448154_at	0.031	0.18
Col19a1	1421698_a_at	0.024	0.18
Ptprg	1429691_at	0.076	0.18
Jam2	1419288_at	0.037	0.18
NA	1434632_at	0.041	0.18
Slc4a4	1426432_a_at	0.042	0.18
Cyp39a1	1418780_at	0.024	0.18
Klhl13	1448269_a_at	0.062	0.18
Slc7a2	1422648_at	0.062	0.18
Egfl6	1419332_at	0.040	0.18
Ncam2	1425301_at	0.022	0.18
Tenm3	1449315_at	0.062	0.18
Col11a1	1418599_at	0.058	0.18
Ctnnd2	1422592_at	0.054	0.18
Kcnh8	1440980_at	0.042	0.18
Rsph9	1424763_at	0.021	0.18
Id4	1438441_at	0.076	0.17
Angpt2	1448831_at	0.022	0.17
Tlcd1	1452132_at	0.019	0.17
Arc	1418687_at	0.047	0.17
Slc9a3r1	1438116_x_at	0.048	0.17
S100a16	1447676_x_at	0.024	0.17
B3gnt5	1420994_at	0.028	0.17
Col23a1	1440911_at	0.028	0.17
Folh1	1450154_at	0.024	0.17
Col4a4	1440250_at	0.059	0.17
Nkain4	1422596_at	0.021	0.17
NA	1442257_at	0.029	0.17
Atp1a2	1455136_at	0.023	0.17
Tmtc2	1429809_at	0.023	0.17
Fam196b	1442379_at	0.025	0.17
Kank1	1433742_at	0.023	0.17
Atp1a2	1452308_a_at	0.093	0.17
NA	1441065_at	0.070	0.17
Prima1	1435125_at	0.054	0.16
Rarres2	1428538_s_at	0.035	0.16
Lsamp	1455636_at	0.042	0.16
Gm2115	1456523_at	0.032	0.16
A730056I06Rik	1439894_at	0.024	0.16
Hmgcs2	1423858_a_at	0.024	0.16
Gm14057	1430286_s_at	0.059	0.16
Cyp2j6	1417952_at	0.023	0.16
Dclk1	1424271_at	0.027	0.16
Cyp2j9	1424677_at	0.027	0.16

Tspan18	1442174_at	0.022	0.16
Metrn	1427100_at	0.024	0.16
Efcab12	1441979_at	0.044	0.16
Ppap2b	1448908_at	0.029	0.16
Hmcn1	1438532_at	0.097	0.16
Lgr4	1433891_at	0.023	0.16
Ednrb	1437347_at	0.010	0.16
Rdh5	1418808_at	0.019	0.16
Tlcd1	1436404_at	0.033	0.16
AI464131	1435417_at	0.023	0.16
Epas1	1435436_at	0.025	0.16
Acot1	1449065_at	0.020	0.16
Tmem47	1420514_at	0.022	0.16
Sema3g	1435361_at	0.035	0.15
Celsr1	1418925_at	0.017	0.15
Acot2	1422997_s_at	0.021	0.15
Slc9a3r1	1450982_at	0.020	0.15
Dclk1	1436659_at	0.029	0.15
Nr2f1	1418157_at	0.024	0.15
Sparcl1	1416114_at	0.057	0.15
Hmgcs2	1431833_a_at	0.013	0.15
Ptn	1448254_at	0.024	0.15
Kcnn4	1421038_a_at	0.021	0.15
Gpr126	1437409_s_at	0.022	0.15
Tmem40	1424966_at	0.025	0.15
Daam2	1455717_s_at	0.024	0.15
Gpd1	1448249_at	0.030	0.15
Abat	1433855_at	0.024	0.15
NA	1455603_at	0.031	0.15
Itih5	1441946_at	0.070	0.15
Slc9a3r1	1438115_a_at	0.044	0.15
Bambi	1423753_at	0.025	0.15
Zfp191	1426896_at	0.017	0.15
Mboat2	1425029_a_at	0.020	0.15
Angptl4	1453410_at	0.063	0.15
Mamdc2	1453152_at	0.078	0.15
Mcc	1438081_at	0.042	0.15
Tcf7l1	1450117_at	0.054	0.15
Casp12	1449297_at	0.053	0.15
Gpm6b	1425942_a_at	0.021	0.15
Lpar1	1448606_at	0.044	0.15
Enpp1	1440339_at	0.025	0.15
Cyr61	1416039_x_at	0.017	0.15
Cmtm5	1430600_at	0.019	0.15

Cyp4f15	1449316_at	0.023	0.14
Tmem229a	1434136_at	0.020	0.14
Sox2ot	1460587_at	0.010	0.14
Pex11a	1449442_at	0.027	0.14
Cdo1	1448842_at	0.013	0.14
Slc1a3	1452031_at	0.062	0.14
Crispld1	1423352_at	0.047	0.14
Fads2	1419031_at	0.020	0.14
Asrgl1	1443960_at	0.081	0.14
Angptl4	1417130_s_at	0.022	0.14
Zfpm2	1449314_at	0.030	0.14
Ltbp2	1418061_at	0.023	0.14
Gpc6	1428774_at	0.088	0.14
Aspa	1418472_at	0.024	0.14
Cyp2d22	1419040_at	0.028	0.14
F3	1417408_at	0.039	0.14
Id4	1423259_at	0.024	0.14
S1pr3	1437173_at	0.028	0.13
Lhfp	1433776_at	0.025	0.13
Tbx2	1422545_at	0.019	0.13
Crabp1	1448326_a_at	0.030	0.13
Cyr61	1438133_a_at	0.022	0.13
Adamts5	1422561_at	0.036	0.13
Ppargc1a	1460336_at	0.043	0.13
Lpar1	1417143_at	0.017	0.13
Matn4	1418464_at	0.023	0.13
Cyp2j6	1440691_at	0.013	0.13
Slc12a2	1417623_at	0.024	0.13
2310022B05Rik	1428910_at	0.020	0.12
Tex40	1449074_at	0.022	0.12
Tyrp1	1415862_at	0.024	0.12
Pex11a	1419365_at	0.023	0.12
Ntrk2	1420838_at	0.018	0.12
Sostdc1	1460250_at	0.021	0.12
Bai3	1454782_at	0.030	0.12
Cyp4f14	1419559_at	0.022	0.12
Gsta4	1416368_at	0.017	0.12
Slc7a2	1436555_at	0.022	0.12
Adamts5	1456404_at	0.021	0.12
Id4	1450928_at	0.036	0.12
Ednrb	1423594_a_at	0.020	0.12
Gm266	1436115_at	0.061	0.12
Bcan	1441899_x_at	0.022	0.11
C130071C03Rik	1435179_at	0.058	0.11

Lrrtm1	1452624_at	0.013	0.11
Col11a1	1449154_at	0.091	0.11
Aldoc	1424714_at	0.024	0.11
Jam2	1449408_at	0.022	0.11
Id3	1416630_at	0.027	0.11
Tyrp1	1439409_x_at	0.025	0.11
Car13	1421308_at	0.021	0.11
Copg2os2	1427320_at	0.017	0.11
Fabp7	1450779_at	0.033	0.11
Lrrtm1	1455883_a_at	0.054	0.11
Slc12a2	1448780_at	0.021	0.11
Fzd8	1445815_at	0.044	0.11
Lrrc4c	1437201_at	0.048	0.11
Nrarp	1417985_at	0.017	0.11
Slc4a4	1434096_at	0.022	0.11
Postn	1423606_at	0.022	0.11
Ntrk2	1435196_at	0.020	0.10
Sfrp5	1436075_at	0.010	0.10
Mfsd2a	1428223_at	0.022	0.10
Slc43a3	1422788_at	0.020	0.10
Mlc1	1448139_at	0.024	0.10
Pcdh10	1438134_at	0.076	0.10
Itih5	1436755_at	0.020	0.10
Gja1	1438650_x_at	0.022	0.10
Ptn	1416211_a_at	0.022	0.10
Nfe2l3	1417520_at	0.031	0.10
Vwc2	1455882_x_at	0.082	0.10
Gpr37l1	1424146_at	0.019	0.10
Slitrk2	1441127_at	0.023	0.09
Rbp1	1448754_at	0.081	0.09
Ndnf	1460465_at	0.025	0.09
Copg2os2	1427123_s_at	0.050	0.09
Atp1a2	1427465_at	0.019	0.09
Gja1	1437992_x_at	0.021	0.09
Lgr5	1450988_at	0.022	0.09
Prss35	1434195_at	0.021	0.09
Ptgfrn	1434891_at	0.021	0.09
Fhdc1	1439018_at	0.048	0.09
Slc35f1	1436719_at	0.023	0.09
Ackr3	1417625_s_at	0.023	0.09
Cybrd1	1460604_at	0.042	0.09
Mmd2	1438654_x_at	0.032	0.09
Ttyh1	1422694_at	0.010	0.09
Megf10	1429841_at	0.022	0.08

Atp1a2	1434893_at	0.017	0.08
Cdh11	1450757_at	0.029	0.08
Tmem47	1449885_at	0.023	0.08
Fmo1	1417429_at	0.023	0.08
Mmd2	1424534_at	0.023	0.07
Elovl2	1416444_at	0.021	0.07
Tyrp1	1415861_at	0.019	0.07
Ttyh1	1422695_at	0.081	0.07
Ptprz1	1452284_at	0.021	0.07
Jam2	1436568_at	0.031	0.07
Rassf10	1457140_s_at	0.063	0.07
Hes5	1456010_x_at	0.083	0.07
Pcdh10	1430667_at	0.014	0.07
Aldoc	1451461_a_at	0.017	0.07
Gja1	1438945_x_at	0.020	0.07
Gja1	1415800_at	0.019	0.07
Spon1	1451342_at	0.037	0.06
Fbln2	1423407_a_at	0.017	0.06
Fbln5	1416164_at	0.010	0.06
Hey2	1418106_at	0.023	0.06
Ptprz1	1427019_at	0.019	0.06
Acsbg1	1422428_at	0.010	0.06
Plscr2	1448961_at	0.020	0.06
Lect1	1460258_at	0.027	0.06
NA	1441389_at	0.022	0.06
Rlbp1	1418310_a_at	0.022	0.05
Ptprz1	1418690_at	0.019	0.04
2900052N01Rik	1436231_at	0.022	0.04
Bcan	1416718_at	0.010	0.04
Aqp4	1434449_at	0.022	0.04
Ripk4	1418488_s_at	0.027	0.04
