# Supplementary material for: Differential Transcriptional Profiling of Damaged and Intact Adjacent Dorsal Root Ganglia Neurons in Neuropathic Pain
Source: PLoS One. 2015 Apr 16;10(4):e0123342. doi: 10.1371/journal.pone.0123342 (PMC4400143; doi:10.1371/journal.pone.0123342)
Supplement: S3 Table — Analysis of microarray results. Included are all genes with p < 0.1 and fold change >2. (n = 3, two-way ANOVA with Benjamini-Hochberg correction). (PDF) [file pone.0123342.s004.pdf]

Gene	*probe ID*	*adjusted p*	*fold change*
Serpinb1a	1448301_s_at	0.081	11.77
Shisa9	1435424_x_at	0.053	11.74
Sox11	1453125_at	0.051	10.84
Sox11	1429372_at	0.056	10.49
NA	1441815_at	0.063	9.35
NA	1446324_at	0.051	9.06
Inhbb	1426858_at	0.098	8.52
NA	1439990_at	0.062	8.09
Gpr151	1457555_at	0.070	7.82
Sox11	1429051_s_at	0.073	7.70
Gna14	1447791_s_at	0.056	7.32
Mmp16	1440161_at	0.056	7.23
Sox11	1453002_at	0.064	7.09
3632451O06Rik	1450770_at	0.056	6.99
NA	1431225_at	0.071	6.67
C78859	1420048_at	0.074	6.33
Gna14	1420385_at	0.056	5.90
Lmo7	1455056_at	0.082	5.74
Sez6l	1424764_at	0.067	5.60
Pcsk2	1447992_s_at	0.070	5.14
Npy2r	1417489_at	0.087	5.10
Sertm1	1436444_at	0.050	5.02
Akap6	1440859_at	0.083	4.69
Slc6a19	1428595_at	0.091	4.67
Tes	1424246_a_at	0.051	4.63
Adam8	1416871_at	0.074	4.61
Elmod1	1434083_a_at	0.065	4.57
Thy1	1423135_at	0.062	4.48
Fgf3	1441914_x_at	0.100	4.42
Cacna2d1	1425861_x_at	0.057	4.37
P2rx3	1458396_at	0.096	4.29
D430019H16Rik	1455447_at	0.068	4.28
Qrfpr	1457048_at	0.056	4.28
Gap43	1423537_at	0.071	4.23
Mctp2	1457707_at	0.087	4.17
Stmn4	1418105_at	0.098	4.14
NA	1439265_at	0.097	4.08
Pcsk2	1428305_at	0.062	4.06
Esd	1438488_at	0.072	3.96
Pappa2	1444451_at	0.074	3.93
Lynx1	1441952_x_at	0.079	3.88
NA	1436463_at	0.070	3.86
Gnpnat1	1423158_at	0.089	3.82

St8sia1	1455695_at	0.069	3.78
Meg3	1428765_at	0.074	3.77
Sh3yl1	1449579_at	0.087	3.75
Gal	1460668_at	0.050	3.74
1110046J04Rik	1457779_at	0.056	3.62
Stmn2	1423281_at	0.056	3.58
Onecut2	1460044_at	0.096	3.57
Plxna4	1457840_at	0.074	3.53
Mfhas1	1429005_at	0.095	3.45
Cers6	1434418_at	0.071	3.38
2810037O22Rik	1428866_at	0.073	3.37
Pde7a	1438041_at	0.093	3.36
Hhipl1	1430062_at	0.062	3.36
Miat	1455325_at	0.074	3.35
Galnt9	1434055_at	0.056	3.35
Prokr2	1437695_at	0.062	3.34
Jakmip2	1436552_at	0.079	3.33
St8sia3	1440029_at	0.081	3.30
Slc41a2	1452445_at	0.070	3.28
Tnik	1455256_at	0.062	3.24
Ina	1448991_a_at	0.087	3.20
Wisp1	1448594_at	0.082	3.11
Ppfia2	1456856_at	0.083	3.11
Elovl7	1440354_at	0.056	3.09
Vash2	1451105_at	0.062	3.08
Tecta	1419632_at	0.056	3.06
Clvs1	1459941_at	0.076	3.05
Snhg11	1434292_at	0.074	3.05
Arhgap12	1451526_at	0.096	3.05
0610010F05Rik	1428652_at	0.087	3.02
NA	1456593_at	0.074	2.98
Cacna2d1	1440397_at	0.059	2.98
Cd109	1436346_at	0.074	2.97
A830010M20Rik	1436117_at	0.051	2.97
Gpr85	1424896_at	0.051	2.96
Arg2	1418847_at	0.083	2.93
Them4	1427055_at	0.096	2.93
Meg3	1429256_at	0.099	2.93
Rnf11	1426405_at	0.062	2.92
Aldh1l2	1436119_at	0.067	2.91
Nrip1	1434384_at	0.087	2.90
Rab33a	1417529_at	0.056	2.86
Zbtb46	1429168_at	0.069	2.84
1700025G04Rik	1455732_at	0.056	2.84

B3galt1	1455234_at	0.083	2.82
Gpr85	1437618_x_at	0.082	2.81
Zwint	1427540_at	0.065	2.80
Gna14	1449848_at	0.082	2.73
Nup93	1424291_at	0.056	2.72
Rian	1427580_a_at	0.092	2.72
Pde1c	1436251_at	0.086	2.70
Stau2	1425534_at	0.087	2.68
Rsad1	1437449_at	0.056	2.68
Ank3	1447259_at	0.079	2.67
Ano4	1442143_at	0.087	2.66
Cacna2d1	1433643_at	0.063	2.65
Rasd2	1427344_s_at	0.067	2.65
Onecut2	1440005_at	0.088	2.63
Nsg1	1423055_at	0.087	2.63
Apc	1435543_at	0.056	2.63
Mtus2	1434780_at	0.091	2.62
Phactr2	1455101_at	0.051	2.62
Rab39b	1435014_at	0.056	2.56
Usp13	1455473_at	0.062	2.56
Fbxw2	1424884_at	0.096	2.55
Tes	1460378_a_at	0.062	2.55
Sgtb	1437977_at	0.065	2.54
Unc13c	1455304_at	0.068	2.54
NA	1441885_s_at	0.087	2.53
Hn1	1416028_a_at	0.098	2.53
Gfra3	1418880_at	0.081	2.53
Zwint	1427539_a_at	0.079	2.52
NA	1445567_at	0.056	2.49
Cadm3	1418922_at	0.062	2.48
Srl	1436867_at	0.064	2.47
Setd4	1460373_a_at	0.062	2.45
Ppbp	1418480_at	0.056	2.40
Col24a1	1453418_at	0.089	2.39
Nrip1	1449089_at	0.071	2.38
Meg3	1452183_a_at	0.071	2.37
A830039N20Rik	1455554_at	0.067	2.35
B3galt1	1441396_at	0.060	2.35
Stmn3	1460181_at	0.098	2.34
Reep1	1433509_s_at	0.089	2.33
Gng3	1417428_at	0.064	2.33
9630033F20Rik	1439859_at	0.087	2.32
AA467197	1434046_at	0.051	2.31
B4galnt4	1435913_at	0.065	2.30

Lppr1	1436733_at	0.095	2.30
Polr3k	1422752_at	0.098	2.30
Mtss1	1424826_s_at	0.087	2.30
Meg3	1439380_x_at	0.086	2.28
Cct4	1430034_at	0.065	2.27
Slc6a19	1455442_at	0.086	2.27
Cep170	1437154_at	0.098	2.26
Sez6l2	1434641_x_at	0.087	2.26
Cnst	1436795_at	0.080	2.25
Nkain1	1449553_at	0.065	2.25
Prkaa2	1434766_at	0.056	2.23
Stx1a	1437390_x_at	0.051	2.23
NA	1445148_at	0.093	2.22
Psmd11	1440253_at	0.089	2.22
Smad1	1448208_at	0.051	2.22
Draxin	1456158_at	0.098	2.21
Ipw	1440557_at	0.090	2.21
Syt4	1415844_at	0.093	2.19
Cda	1427357_at	0.081	2.19
Ube2k	1417188_s_at	0.056	2.18
Ugcg	1435133_at	0.085	2.18
Elavl3	1435871_at	0.065	2.17
Ufl1	1429008_at	0.070	2.16
Glce	1428374_at	0.070	2.16
NA	1442913_at	0.062	2.16
Glrx3	1447868_x_at	0.089	2.16
38596.00	1452357_at	0.086	2.15
Rapgef4	1425518_at	0.070	2.15
D10Bwg1379e	1455995_at	0.074	2.15
Sycp2	1444122_at	0.095	2.15
NA	1440003_at	0.056	2.13
2810416G20Rik	1435171_at	0.056	2.12
Mtmr4	1459874_s_at	0.087	2.12
Trmt5	1452086_at	0.062	2.12
Zwint	1429787_x_at	0.065	2.11
Cyp26a1	1419430_at	0.062	2.11
Faxc	1437753_at	0.087	2.11
Mllt11	1416313_at	0.081	2.11
Casp3	1449839_at	0.056	2.10
Sphkap	1454926_at	0.094	2.10
Agfg1	1426922_s_at	0.062	2.09
2010012O05Rik	1439561_at	0.087	2.08
NA	1438864_at	0.095	2.08
Chst1	1449147_at	0.067	2.07

NA	1442557_at	0.082	2.07
Thsd7a	1456130_at	0.056	2.07
5830428H23Rik	1428748_at	0.056	2.07
Zfp938	1455775_at	0.070	2.07
Fam131b	1455421_x_at	0.100	2.06
Rpl31	1420381_a_at	0.064	2.06
Asphd1	1456837_at	0.068	2.05
Ttc37	1435568_at	0.083	2.05
Sptbn2	1452269_at	0.056	2.05
Amer2	1454051_at	0.066	2.04
Myadm	1439389_s_at	0.071	2.03
Zfp655	1429217_at	0.074	2.03
Fam102b	1434828_at	0.098	2.02
Cyb5r1	1424048_a_at	0.073	2.02
Hn1	1438988_x_at	0.089	2.01
6430709C05Rik	1432713_at	0.076	2.01
NA	1438331_at	0.087	2.01
Slc29a4	1441902_x_at	0.096	2.00
Slc27a1	1422811_at	0.069	0.50
Rassf10	1453201_at	0.089	0.50
Acadm	1415984_at	0.087	0.50
Sort1	1450955_s_at	0.087	0.50
Bsg	1456616_a_at	0.062	0.50
Pfkm	1416780_at	0.067	0.50
Smo	1427048_at	0.073	0.50
Dad1	1418528_a_at	0.077	0.50
Pnkd	1418746_at	0.087	0.50
Tmem97	1416376_at	0.087	0.49
Chpt1	1435446_a_at	0.064	0.49
H2afv	1436596_at	0.060	0.49
Gas1	1416855_at	0.093	0.49
Cdk6	1435338_at	0.074	0.49
Vim	1438118_x_at	0.071	0.48
Lrrc8d	1433506_at	0.070	0.48
Sh3pxd2b	1435644_at	0.088	0.48
Ier2	1416442_at	0.070	0.48
Rgcc	1438511_a_at	0.094	0.48
Pon2	1429019_s_at	0.082	0.48
Hsd17b11	1434642_at	0.098	0.48
Ctnna1	1448149_at	0.079	0.48
Ctnnbip1	1417567_at	0.077	0.48
Acvr2a	1451004_at	0.085	0.48
Ctnnal1	1420930_s_at	0.096	0.47

Vamp5	1430522_a_at	0.062	0.47
Phlda2	1417837_at	0.056	0.47
Ripk4	1418487_at	0.067	0.47
Dbi	1455976_x_at	0.087	0.47
1190002N15Rik	1433582_at	0.079	0.46
NA	1455418_at	0.087	0.46
Olfml2b	1423915_at	0.094	0.46
Calca	1452004_at	0.098	0.46
Btbd11	1459838_s_at	0.067	0.46
Fdps	1423418_at	0.089	0.46
Insc	1453259_at	0.087	0.45
Cdh20	1450312_at	0.062	0.45
Eci2	1431012_a_at	0.065	0.45
Pmepa1	1422705_at	0.065	0.45
Zfp503	1423835_at	0.098	0.45
Hdac4	1454693_at	0.085	0.44
Klhl13	1416242_at	0.087	0.44
Fbxl7	1456220_at	0.087	0.44
Stard4	1455011_at	0.098	0.44
Dag1	1426778_at	0.099	0.44
Dbi	1438093_x_at	0.060	0.44
Laptm4b	1416148_at	0.067	0.44
Lss	1420013_s_at	0.087	0.44
Rnf215	1452756_at	0.062	0.44
Elovl6	1417404_at	0.089	0.44
Hspa12a	1436961_at	0.095	0.43
Abhd3	1417946_at	0.093	0.43
Sardh	1448426_at	0.081	0.43
Smpd2	1416999_at	0.098	0.43
Il17rd	1429893_at	0.062	0.43
Oat	1416452_at	0.056	0.42
Celsr1	1418925_at	0.081	0.42
Spry4	1445669_at	0.094	0.42
Pde4d	1435280_at	0.098	0.42
Rap1gap	1428443_a_at	0.062	0.42
Rhobtb3	1433647_s_at	0.087	0.41
Gramd1b	1435229_at	0.086	0.41
Fads1	1423680_at	0.059	0.41
Dbi	1433991_x_at	0.056	0.41
Ldhb	1455235_x_at	0.067	0.41
Pla2g16	1451611_at	0.071	0.41
Ripk1	1419508_at	0.057	0.41
Ddhd1	1454070_a_at	0.088	0.41
Dag1	1426779_x_at	0.080	0.41

A030001O10Rik	1434989_at	0.098	0.41
Gm2115	1442595_at	0.058	0.40
Pnmal2	1435960_at	0.056	0.40
Pde4b	1422473_at	0.093	0.40
Aldh1l1	1424400_a_at	0.055	0.40
Bcl2	1457687_at	0.056	0.40
Ptges	1439747_at	0.079	0.40
Plxnb3	1418750_at	0.051	0.40
Ncald	1417568_at	0.070	0.39
Hes1	1418102_at	0.070	0.39
Zfp191	1426895_at	0.062	0.39
Fnta	1417465_at	0.060	0.39
Scarb1	1416050_a_at	0.057	0.39
Dag1	1423872_a_at	0.091	0.39
Adhfe1	1424393_s_at	0.087	0.38
Ttyh1	1426617_a_at	0.064	0.38
Lrrc4	1416097_at	0.091	0.38
Ift122	1441259_s_at	0.082	0.38
Gpr125	1426782_at	0.062	0.38
Itgb8	1436223_at	0.089	0.38
Btbd3	1425660_at	0.081	0.38
Add3	1423297_at	0.075	0.38
Cmtm5	1429798_s_at	0.070	0.37
Elovl5	1437211_x_at	0.089	0.37
Smpdl3a	1416635_at	0.062	0.37
Hmgcr	1427229_at	0.087	0.37
Slc4a4	1421225_a_at	0.056	0.37
Tnxb	1450798_at	0.081	0.37
Prodh	1417629_at	0.056	0.37
Nlgn3	1436135_at	0.098	0.37
Ldhb	1416183_a_at	0.064	0.37
1190002N15Rik	1433581_at	0.078	0.36
Amot	1454890_at	0.099	0.36
Plp1	1425468_at	0.078	0.36
Ptprt	1439725_at	0.081	0.36
Ephx1	1422438_at	0.067	0.36
Vim	1456292_a_at	0.078	0.36
Lysmd2	1428626_at	0.075	0.36
Fjx1	1450728_at	0.077	0.36
Btbd3	1433868_at	0.081	0.36
Ldhb	1448237_x_at	0.074	0.36
Lphn3	1460440_at	0.076	0.36
Chadl	1440958_at	0.056	0.36
NA	1436092_at	0.095	0.36

Scd1	1415964_at	0.062	0.35
Kit	1452514_a_at	0.089	0.35
Grb14	1417673_at	0.098	0.35
Rnd2	1422670_at	0.073	0.35
Igfbp4	1423756_s_at	0.081	0.35
Eepd1	1417877_at	0.056	0.35
Socs3	1416576_at	0.074	0.35
Micall2	1434322_at	0.098	0.34
Plp1	1425467_a_at	0.062	0.34
Mxi1	1450376_at	0.062	0.34
Mest	1423294_at	0.062	0.34
Casp12	1418981_at	0.091	0.34
Prnp	1416130_at	0.087	0.34
Col23a1	1429209_at	0.095	0.34
Slc12a2	1417622_at	0.056	0.34
Mpzl1	1428168_at	0.093	0.34
C1qtnf5	1424762_at	0.089	0.34
Bcl2	1437122_at	0.082	0.34
Add3	1426574_a_at	0.086	0.34
Prex2	1436569_at	0.074	0.33
Dag1	1456131_x_at	0.091	0.33
40057.00	1417038_at	0.083	0.33
Psph	1415673_at	0.079	0.33
Plscr1	1453181_x_at	0.070	0.33
Atp1a2	1443823_s_at	0.081	0.33
Slc12a4	1417446_at	0.081	0.33
Pla2g16	1445597_s_at	0.062	0.33
Tmod2	1431326_a_at	0.081	0.33
Kcnc4	1425090_s_at	0.092	0.33
Plp1	1451718_at	0.062	0.33
Tlcd1	1452132_at	0.061	0.32
Dhh	1434959_at	0.062	0.32
Elovl6	1417403_at	0.084	0.32
Kirrel	1434797_at	0.096	0.32
Pde12	1454963_at	0.074	0.32
Myo16	1445532_at	0.074	0.32
Abca8a	1427371_at	0.086	0.32
Kif1b	1455182_at	0.087	0.32
Dbi	1422432_at	0.068	0.32
Lrrn3	1434539_at	0.065	0.32
Gm14005	1444524_at	0.100	0.31
Pdpn	1419309_at	0.073	0.31
Slc22a17	1448209_a_at	0.067	0.31
Cdh15	1418602_at	0.070	0.31

Trib2	1426640_s_at	0.079	0.31
Tenm3	1429178_at	0.087	0.31
Dpyd	1427945_at	0.099	0.30
Spry2	1436584_at	0.056	0.30
Rdh5	1418808_at	0.060	0.30
Dpyd	1427946_s_at	0.067	0.30
Hs3st1	1423450_a_at	0.099	0.30
Fam198b	1429637_at	0.098	0.30
Gpr126	1437408_at	0.062	0.30
Fam19a5	1419489_at	0.098	0.30
Crym	1416776_at	0.056	0.29
Slc4a4	1452071_at	0.056	0.29
A730056I06Rik	1439894_at	0.091	0.29
Cyp4f15	1449316_at	0.093	0.29
Trim2	1448551_a_at	0.090	0.29
Lgi4	1434121_at	0.067	0.29
Acaa2	1428146_s_at	0.056	0.29
Kcnj10	1419601_at	0.098	0.29
Frmd6	1451264_at	0.059	0.29
Fam107a	1434202_a_at	0.090	0.29
Fam196b	1442379_at	0.092	0.29
Pcx	AFFX-PyruCarbMur/L09192_3_at	0.067	0.29
Emp2	1433670_at	0.087	0.29
Cyp2d22	1419039_at	0.069	0.29
AI464131	1435417_at	0.087	0.29
Lama4	1424807_at	0.093	0.28
Htra1	1416749_at	0.098	0.28
Mgst1	1415897_a_at	0.070	0.28
Rsph9	1424763_at	0.062	0.28
Msmo1	1423078_a_at	0.084	0.28
Plscr4	1433626_at	0.100	0.28
Add3	1423298_at	0.083	0.28
Gpam	1419499_at	0.053	0.28
Zfp191	1426896_at	0.056	0.28
Sox2ot	1460587_at	0.050	0.28
Metrn	1427100_at	0.087	0.28
Atp1b2	1435148_at	0.062	0.28
Sorcs2	1419358_at	0.089	0.28
Ncam1	1450437_a_at	0.070	0.28
Sfrp5	1423023_at	0.062	0.28
Itpr3	1417297_at	0.082	0.28
Fads2	1449325_at	0.060	0.27
Rcn1	1417090_at	0.098	0.27

Col4a1	1426348_at	0.082	0.27
Tmtc2	1429809_at	0.073	0.27
Enpp1	1440339_at	0.098	0.27
Ece1	1455741_a_at	0.087	0.27
Pcdh10	1425563_s_at	0.071	0.27
Frem2	1457038_at	0.074	0.27
B130021B11Rik	1438989_s_at	0.076	0.27
Pcx	1416383_a_at	0.060	0.27
Vim	1450641_at	0.062	0.27
Mboat2	1425029_a_at	0.062	0.27
Mdk	1416006_at	0.067	0.27
Elovl5	1415840_at	0.067	0.27
S100a16	1425560_a_at	0.083	0.27
Ednrb	1423594_a_at	0.065	0.26
Me1	1416632_at	0.074	0.26
Abhd4	1416315_at	0.057	0.26
Gpm6b	1423091_a_at	0.051	0.26
Cdh1	1448261_at	0.062	0.26
Ptpn13	1452127_a_at	0.083	0.26
Gpr126	1437409_s_at	0.073	0.26
Pcsk5	1451406_a_at	0.083	0.26
Ctnnd2	1456116_at	0.062	0.26
Car13	1421307_at	0.056	0.26
Slco3a1	1434537_at	0.074	0.26
Me1	1430307_a_at	0.074	0.26
Tex40	1449074_at	0.077	0.25
NA	1457373_at	0.057	0.25
B130021B11Rik	1437558_at	0.087	0.25
Cdo1	1448842_at	0.051	0.25
Fads2	1443838_x_at	0.087	0.25
NA	1457741_at	0.074	0.25
Sparc	1416589_at	0.063	0.25
Gstk1	1452823_at	0.062	0.25
Sorbs1	1425826_a_at	0.085	0.25
Sox2	1416967_at	0.056	0.24
Dennd2a	1433500_at	0.087	0.24
Angpt2	1448831_at	0.062	0.24
Serpine2	1416666_at	0.060	0.24
Hmgcs2	1431833_a_at	0.051	0.24
B3gnt5	1420994_at	0.081	0.24
Tmem47	1420514_at	0.063	0.24
Ppap2b	1429514_at	0.074	0.24
Ncam2	1425301_at	0.062	0.24
Nkain4	1422596_at	0.056	0.24

Kank1	1433742_at	0.066	0.23
Fzd8	1423348_at	0.071	0.23
Dkk3	1448669_at	0.070	0.23
Tmem40	1424966_at	0.081	0.23
Ramp2	1418187_at	0.082	0.23
Sema3g	1435361_at	0.098	0.23
Cyp4f14	1419559_at	0.072	0.23
Dclk1	1436659_at	0.087	0.23
Cyp39a1	1418780_at	0.065	0.23
Kcnn4	1421038_a_at	0.060	0.23
Cebpd	1423233_at	0.062	0.23
Lurap1l	1428384_at	0.060	0.23
Tspan18	1442174_at	0.060	0.22
Gpd1	1448249_at	0.087	0.22
Epas1	1435436_at	0.073	0.22
Zfpm2	1449314_at	0.092	0.22
Slc12a2	1417623_at	0.086	0.22
S1pr3	1438658_a_at	0.059	0.22
Cmtm5	1430600_at	0.056	0.22
Ptn	1448254_at	0.071	0.22
Cyp2j6	1440691_at	0.051	0.21
Abat	1433855_at	0.070	0.21
Atp1a2	1455136_at	0.062	0.21
Acot1	1449065_at	0.056	0.21
Slc9a3r1	1450982_at	0.056	0.21
Aspa	1418472_at	0.074	0.21
Ptn	1416211_a_at	0.074	0.21
Col23a1	1440911_at	0.070	0.21
Cyp2j6	1417952_at	0.062	0.21
Gbp6	1447927_at	0.061	0.21
Lgr4	1433891_at	0.062	0.21
Fads2	1419031_at	0.056	0.21
Ndrg2	1448154_at	0.070	0.21
Ppap2b	1448908_at	0.074	0.20
Rarres2	1428538_s_at	0.082	0.20
Adamts5	1450658_at	0.064	0.20
Sostdc1	1449340_at	0.055	0.20
Fam102a	1426894_s_at	0.065	0.20
Copg2os2	1427320_at	0.056	0.20
Ednrb	1437347_at	0.046	0.20
Lsamp	1455636_at	0.087	0.20
S100a16	1447676_x_at	0.062	0.20
Id4	1423259_at	0.071	0.20
2310022B05Rik	1428910_at	0.056	0.20

Vwa1	1426399_at	0.090	0.19
Col12a1	1434411_at	0.072	0.19
Matn2	1419442_at	0.086	0.19
Kctd1	1422293_a_at	0.087	0.19
Slc12a2	1448780_at	0.062	0.19
Gsta4	1416368_at	0.051	0.19
Lrrtm1	1452624_at	0.051	0.19
Daam2	1455717_s_at	0.062	0.19
Tmem229a	1434136_at	0.056	0.18
Cyp2j9	1424677_at	0.064	0.18
Sostdc1	1460250_at	0.060	0.18
Arhgef26	1429185_at	0.056	0.18
Nrarp	1417985_at	0.052	0.18
Id3	1416630_at	0.084	0.18
Slc4a4	1434096_at	0.065	0.18
Ndnf	1460465_at	0.087	0.18
Tyrp1	1439409_x_at	0.076	0.18
Tbx2	1422545_at	0.051	0.17
Hmgcs2	1423858_a_at	0.060	0.17
Cyr61	1416039_x_at	0.050	0.17
Lgr5	1450988_at	0.070	0.17
Slc43a3	1422788_at	0.056	0.17
Cyp2d22	1419040_at	0.070	0.17
Jam2	1449408_at	0.062	0.17
NA	1441389_at	0.084	0.17
Car13	1421308_at	0.056	0.17
Acot2	1422997_s_at	0.053	0.17
Elovl2	1416444_at	0.065	0.17
Pcdh10	1430667_at	0.051	0.17
Arc	1418687_at	0.077	0.17
Sfrp5	1436075_at	0.050	0.16
Adamts5	1456404_at	0.056	0.16
Tyrp1	1415862_at	0.065	0.16
Bcan	1441899_x_at	0.062	0.16
Gpm6b	1425942_a_at	0.053	0.16
Ntrk2	1420838_at	0.051	0.16
Ptgfrn	1434891_at	0.062	0.16
Megf10	1429841_at	0.069	0.16
Matn4	1418464_at	0.062	0.16
Gja1	1438650_x_at	0.062	0.15
Lhfp	1433776_at	0.062	0.15
Car2	1448752_at	0.092	0.15
Ntrk2	1435196_at	0.056	0.15
Ttyh1	1422694_at	0.050	0.15

Ackr3	1417625_s_at	0.069	0.15
Fabp7	1450779_at	0.081	0.15
Postn	1423606_at	0.062	0.15
Angptl4	1417130_s_at	0.056	0.15
Gja1	1437992_x_at	0.059	0.14
Cyr61	1438133_a_at	0.056	0.14
Mfsd2a	1428223_at	0.060	0.14
Slitrk2	1441127_at	0.062	0.14
Lpar1	1417143_at	0.050	0.14
Gpr37l1	1424146_at	0.051	0.14
Slc7a2	1436555_at	0.056	0.14
Atp1a2	1427465_at	0.051	0.14
Cdh11	1450757_at	0.083	0.13
Fmo1	1417429_at	0.067	0.13
F3	1417408_at	0.070	0.13
Itih5	1436755_at	0.056	0.13
Rlbp1	1418310_a_at	0.070	0.13
Slc35f1	1436719_at	0.065	0.13
Mlc1	1448139_at	0.062	0.13
Plscr2	1448961_at	0.061	0.13
Atp1a2	1452308_a_at	0.100	0.13
Mmd2	1424534_at	0.066	0.13
Ptprz1	1452284_at	0.056	0.12
Atp1a2	1434893_at	0.051	0.12
Hey2	1418106_at	0.070	0.12
Prss35	1434195_at	0.056	0.12
Gja1	1438945_x_at	0.056	0.11
Ptprz1	1427019_at	0.056	0.11
Acsbg1	1422428_at	0.046	0.11
S1pr3	1437173_at	0.057	0.11
Aldoc	1451461_a_at	0.051	0.11
Adamts5	1422561_at	0.062	0.11
Tyrp1	1415861_at	0.055	0.11
Lect1	1460258_at	0.083	0.11
Tmem47	1449885_at	0.060	0.10
Fbln2	1423407_a_at	0.051	0.10
Gja1	1415800_at	0.051	0.09
2900052N01Rik	1436231_at	0.062	0.09
Fbln5	1416164_at	0.046	0.08
Ptprz1	1418690_at	0.053	0.07
Bcan	1416718_at	0.046	0.07
Aqp4	1434449_at	0.057	0.05
